# Unraveling the Microbiome–Human Body Axis: A Comprehensive Examination of Therapeutic Strategies, Interactions and Implications

**DOI:** 10.3390/ijms25105561

**Published:** 2024-05-20

**Authors:** Gabriel Olteanu, Maria-Alexandra Ciucă-Pană, Ștefan Sebastian Busnatu, Dumitru Lupuliasa, Sorinel Marius Neacșu, Magdalena Mititelu, Adina Magdalena Musuc, Corina-Bianca Ioniță-Mîndrican, Steluța Constanța Boroghină

**Affiliations:** 1 Department of Clinical Laboratory and Food Safety, Faculty of Pharmacy, University of Medicine and Pharmacy Carol Davila, 020956 Bucharest, Romania; gabriel.olteanu@mst.umfcd.ro; 2 Department of Cardiology, Carol Davila University of Medicine and Pharmacy, Bagdasar-Arseni Emergency Hospital, 050474 Bucharest, Romania; maria.alexandra.pana@drd.umfcd.ro; 3 Department of Cardio-Thoracic Pathology, Faculty of Medicine, Carol Davila University of Medicine and Pharmacy, 050474 Bucharest, Romania; stefan.busnatu@umfcd.ro; 4 Department of Pharmaceutical Technology and Bio-Pharmacy, Faculty of Pharmacy, Carol Davila University of Medicine and Pharmacy, 020945 Bucharest, Romania; dumitru.lupuliasa@umfcd.ro (D.L.); sorinel-marius.neacsu@drd.umfcd.ro (S.M.N.); 5 Institute of Physical Chemistry—Ilie Murgulescu, Romanian Academy, 060021 Bucharest, Romania; 6 Department of Toxicology, Faculty of Pharmacy, Carol Davila University of Medicine and Pharmacy, 020945 Bucharest, Romania; corina-bianca.ionita-mindrican@drd.umfcd.ro; 7 Department of Complementary Sciences, History of Medicine and Medical Culture, Carol Davila University of Medicine and Pharmacy, 050474 Bucharest, Romania; steluta.boroghina@umfcd.ro

**Keywords:** intestinal microbiota, probiotics, short-chain fatty acids, microbiota fecal transplantation, dysbiosis

## Abstract

This review scrutinizes the intricate interplay between the microbiome and the human body, exploring its multifaceted dimensions and far-reaching implications. The human microbiome, comprising diverse microbial communities inhabiting various anatomical niches, is increasingly recognized as a critical determinant of human health and disease. Through an extensive examination of current research, this review elucidates the dynamic interactions between the microbiome and host physiology across multiple organ systems. Key topics include the establishment and maintenance of microbiota diversity, the influence of host factors on microbial composition, and the bidirectional communication pathways between microbiota and host cells. Furthermore, we delve into the functional implications of microbiome dysbiosis in disease states, emphasizing its role in shaping immune responses, metabolic processes, and neurological functions. Additionally, this review discusses emerging therapeutic strategies aimed at modulating the microbiome to restore host–microbe homeostasis and promote health. Microbiota fecal transplantation represents a groundbreaking therapeutic approach in the management of dysbiosis-related diseases, offering a promising avenue for restoring microbial balance within the gut ecosystem. This innovative therapy involves the transfer of fecal microbiota from a healthy donor to an individual suffering from dysbiosis, aiming to replenish beneficial microbial populations and mitigate pathological imbalances. By synthesizing findings from diverse fields, this review offers valuable insights into the complex relationship between the microbiome and the human body, highlighting avenues for future research and clinical interventions.

## 1. Introduction

Lifestyle and, especially, eating habits profoundly influence the communities of microorganisms which exist in the human body, i.e., the microbiome [1,2]. Bacteria, fungi, Archaea, viruses, and protozoa are all part of the microorganism population that can be found in a region, known as the microbiota [3,4]. The origin of the term “microbiota” dates back to 1900. Various microorganisms (such as bacteria, fungi, viruses, protozoa) coexist in different regions of the human body (spanning the gut, oral cavity, skin, lungs, both upper and lower respiratory tract and vaginal mucosa) [5,6,7,8,9,10,11,12,13,14,15]. The microbiota present in the oral cavity is notably recognized as the second-largest microbial community within the human body, with the gut microbiota being the largest [10]. Moreover, the human microbiota, often referred to as the “hidden organ” or “forgotten organ”, imparts over 150-fold more genetic information than the entire human genome [16,17]. Although the terms “microbiome” and “microbiota” are frequently interchanged, there are certain distinctions between them. The microbiota describes the living microorganisms in a specific environment. On the other hand, the microbiome refers to the collective genomes of all microorganisms in a particular environment. This includes not only the community of microorganisms but also their microbial structural elements (various physical components like cell walls, membranes, biofilms, extracellular polymeric substances, flagella, and pili or fimbriae), metabolites, and the environmental conditions [9]. So, the microbiome encompasses a broader spectrum than that of the microbiota and can be considered an umbrella term [9,18].

**Figure 1:**
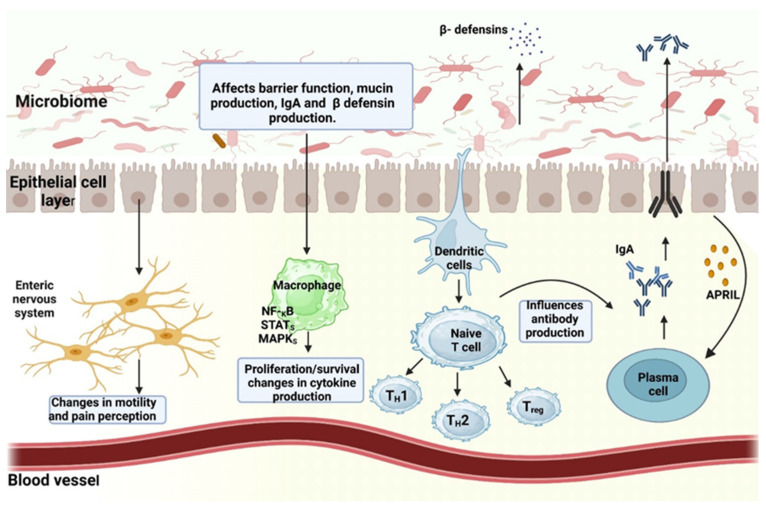
The gut microbiome and its effects on the intestinal mucosa. Legend: IgA (immunoglobulin A); NF-κB (nuclear factor-kappa B); STAT (Signal Transducer and Activator of Transcription); MAPK (Mitogen-Activated Protein Kinase). Created with BioRender.com (accessed on 15 November 2023).

Beyond microbial biodiversity, a healthy gut microbiome can be defined by the presence of classes of microorganisms that improve metabolism, resistance to infectious agents and inflammation, resistance to cancer or autoimmunity, endocrine signaling, and brain function (brain–gut axis). It appears that the microbiome can mediate these effects through the secretion of factors that modulate intestinal permeability (Figure 1), the mucosal layer, epithelial cell function, innate and adaptive immunity, intestinal motility, and neurotransmission [6].

The gastrointestinal microbiota holds significant importance in facilitating digestion and the assimilation of nutrients. Specific bacterial strains and Archaea (genus *Methanobrevibacter*) assist in the breakdown of dietary components that the human body alone cannot metabolize, thereby generating crucial nutrients and vitamins. A harmonized microbiota is pivotal for sustaining a robust immune system by orchestrating immune responses, educating immune cells, and defending against harmful pathogens. Perturbations in this microbial equilibrium can contribute to autoimmune ailments and increase vulnerability to infections [19,20,21].

An aberrant microbiota composition can incite chronic inflammation, which correlates with a spectrum of diseases, including inflammatory bowel diseases (IBD), cardiovascular disorders, and certain cancers [22]. Investigating the distinct roles played by diverse microbial species within the microbiota remains an active field of study. Variables such as dietary habits, lifestyle choices, antibiotic utilization, and environmental factors wield influence over the composition and diversity of the microbiota [23].

Efforts aimed at modulating the microbiota to enhance overall well-being encompass interventions like probiotics (beneficial live microorganisms), prebiotics (substances fostering the growth of beneficial microbes), and fecal microbiota transplantation (the transfer of fecal matter from a healthy donor to restore a balanced microbiota) [24,25]. However, despite the established correlation between the microbiota and health, the precise causal mechanisms remain under active investigation. Tailored interventions based on an individual’s microbiota profile hold promise in healthcare, potentially yielding more precise and efficacious treatments for a spectrum of health conditions [26,27].

The bidirectional communication channel, known as the gut–brain axis, links the gut and the brain through intricate biochemical signaling [28]. Evolving research posits that the microbiota exerts influence over emotional states, behavioral patterns, and potentially neurological disorders like depression, anxiety, and autism. Dysbiosis within the microbiota has established associations with metabolic irregularities such as obesity and type 2 diabetes, possibly altering nutrient processing and fat storage mechanisms [29,30].

According to a study carried out in Denmark, involving 123 individuals with normal weight and 169 individuals who were obese, it was identified a group of genes linked to the role of gut microbiome in determining health or disease. These genes were categorized into high gene count (HGC) and low gene count (LGC) [4,31]. According to the research, the group of microorganisms known as the HGC microbiome plays a crucial role in maintaining overall health. Individuals possessing a high gene count microbiome typically exhibit a broader spectrum of microbial metabolic functions and a reduced susceptibility to metabolic disorders and obesity [4,6]. On the other hand, individuals harboring a low gene count microbiome are more prone to an increased prevalence of pro-inflammatory bacteria, such as *Bacteroides* and *Ruminococcus gnavus* [32,33,34].

Based on the MetaHIT Consortium proposal, saprophytic microbiota can be classified into three distinct “enterotypes”, namely: Enterotype 1 shows an increased abundance of *Bacteroides* possessing major saccharolytic potential. Enterotype 2 shows an increased amount of *Prevotella* and a high potential for degradation of mucin-type glycoproteins. Enterotype 3, which is abundant in *Ruminococcus* has a potential for mucin degradation and transport of sugars across the plasma membrane [4,35]. Saprophytic microbiota encompass a consortium of bacteria and diverse microorganisms that establish symbiotic relationships within the human body. This community significantly contributes to essential physiological processes such as digestive functions, immune modulation, and the synthesis of vital vitamins.

Each milliliter of the large intestine contains approximately 10^11^ microbial cells, compared to 10^8^ microbial cells in the small intestine [4,36,37,38,39]. Generally, bacterial populations increase from approximately 10^4–5^ CFU/mL (colony-forming unit) in the duodenum to 10^7–8^ CFU/mL in the distal ileum, where transit is slowed. The proportion of Gram-positive and Gram-negative bacterial species, as well as facultative anaerobic and strictly anaerobic species, increases from proximal to distal segments of the small intestine and colon [6,39,40,41].

In general, the saprophytic intestinal microorganisms are mainly made up of *Bacillota phylum* and *Bacteroidetes* species, with *Actinobacteria* and *Verrucomicrobia* following close behind. Despite this typical composition, the gut’s microorganisms vary in their distribution across genders and among individuals, both in time and location. There is a distinct variation in both the quantity and diversity of bacteria from the esophagus to the rectum. The bacterial count ranges from approximately 10^1^ per gram in the esophagus and stomach to 10^12^ per gram in the colon and the farthest segment of the intestine [4,17].

**Figure 2:**
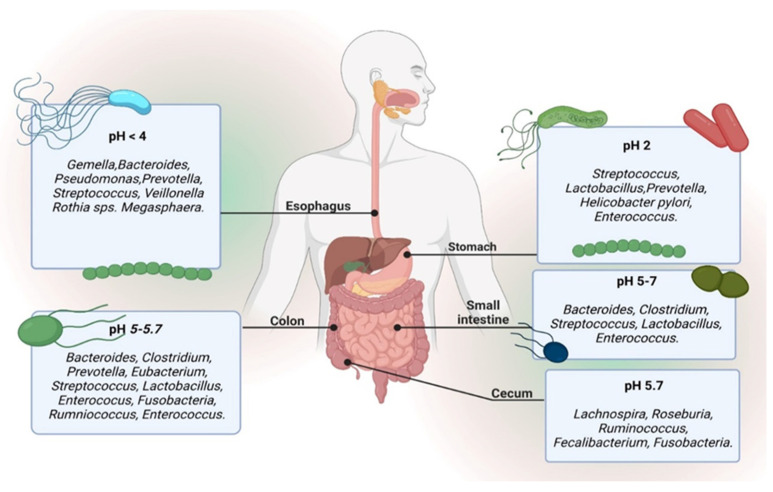
Arrangement of saprophytic bacterial microorganisms within the gastrointestinal tract. Legend: microbiome diversity correlated with pH. Created with BioRender.com (accessed on 15 November 2023).

There is a temporal diversity of saprophytic microorganisms when passing from the esophagus to the distal colon, as can be seen in Figure 2 [42,43,44]. *Streptococcus* emerges as the predominant genus in the distal esophagus, duodenum, and jejunum, while *Helicobacter pylori* takes the lead in the stomach. It can determine the entire reconfiguration of the gastric saprophytic microflora, in the sense that when it occupies the organism as a commensal (*H. pylori*) a rich diversity can be observed in other dominant constituents such as *Streptococcus* (the most dominant), *Prevotella*, *Rothia* and *Veillonella* [42,43,44,45,46]. This diversity changes and decreases when *H. pylori* acquire a pathogenic phenotype. The large intestine harbors over 70% of the microorganisms in the human body, and microbiota is commonly incriminated when discussing disease states, specifically colonic flora. The predominant species inhabiting the large intestine include *Bacillota phylum* and *Bacteroidetes*. The presence of *Proteobacteria* species is significantly low; so, the increased abundance of saprophytic bacteria (*Bacteroides*, *Prevotella*, *Ruminococcus*) associated with the absence of the *Proteobacteria* species suggests a healthy gut microbiota [6].

The primary microbial genera identified in the gut lumen, as illustrated in Figure 2 and detectable in stool samples, comprise *Bacteroides*, *Bifidobacterium*, *Streptococcus*, *Enterobacteriaceae*, *Enterococcus*, *Clostridium*, *Lactobacillus*, and *Ruminococcus*. In contrast, within the mucosa and epithelial crypts of the small intestine, the predominant genera are *Akkermansia*, *Clostridium*, *Enterococcus*, and *Lactobacillus* [47].

The relative proportions of each of these taxa vary significantly inter-individually and even within the same individual during its lifetime [2,15,44,48,49,50].

## 2. Materials and Methods

For an overview of the gut microbiome and the factors that can influence it both positively and negatively, we accessed multiple databases such as Scopus, PubMed, BMC (BioMed Central), medRxiv, PNAS, MDPI, NCBI, and Frontiers, using the following keywords: “microbiome”, “gut microbiota”, “human microbiota”, “microbiome diversity”, “microbiome and disease”, “microbiome and health”, “microbiome and metabolism”, “microbiome and nutrition”, “prebiotics and microbiome”, “dysbiosis”, “host-microbiome interactions”, “microbiome and chronic diseases”, “microbiome and antibiotic use”. The relevant studies were selected, especially from the last two decades (between January 2004 and April 2024). Thus, 245,142 articles were retained, of which over 349 were included in the present study, as follows: 9 articles from the Proceedings of the National Academy of Sciences (PNAS) (Biological Sciences-8, Microbiology-1), 19 articles from BioMed Central (BMC) (Allergy, Asthma & Clinical Immunology-2, Biology-1, BMC Gastroenterology-2, BMC Microbiology-3, Genome Biology-3, Genome Medicine-3, Microbiome-3, Nutrition Journal-2), 4 articles from American Physiological Society, 4 articles from Science Direct, 60 articles from MDPI (Multidisciplinary Digital Publishing Institute), 12 articles from Frontiers (the most cited journal was Frontiers in Cellular and Infection Microbiology-4), two articles from BMJ Journals, one article from medRxiv and 226 articles from NCBI (National Library of Medicine) (most used journals include Nature-13, PloS One-12, Nutrients-9, The American Journal of Clinical Nutrition-6, Gut-5, The ISME Journal-5, World Journal of Gastroenterology-5).

## 3. The Microbiome and Its Implications for Human Health

### 3.1. The Microbiome Nutrition and Lifestyle: A Complex Symbiosis in Intestinal Health

Age seems to play a significant role in shaping the dynamics of the gut microbial ecosystem. Noteworthy shifts in the composition of gut saprophytic microflora during aging are evident, marked by the swift maturation of the microbiome from birth onwards. Additionally, comparisons between the microbial communities of children and adults shed light on these changes [51,52]. Furthermore, dietary preferences have been associated with modifying the microbiome within the digestive system. Large-scale differences (detectable at the bacterial phylum level) were reported between the fecal microbiome of children consuming carbohydrates from plant sources compared to children consuming a Western-type diet [53]. A study that included children from Europe who consumed foods specific to the Western diet and children from Burkina Faso who consumed increased amounts of millet and/or sorghum alongside local plant foods with low lipid and animal protein content demonstrated that the African cohort recorded a significant abundance of *Prevotella* and *Xylanibacter*, and *Shigella* and *Escherichia* were poorly represented [53].

David LA et al. (2013) compared diets centered on vegetable products and those based on animal-derived products to examine alterations in the structural composition of the intestinal microbiome [54]. Consuming a diet that primarily consists of animal products can lead to an increase in the levels of bile-resistant microorganisms such as *Alistipes*, *Bilophila*, and *Bacteroides*. On the other hand, such a diet can cause a decrease in the levels of *Bacillota phylum* that are responsible for breaking down food polysaccharides found in plant products. This indicates that the activity of the microbiota is similar to that of herbivorous and carnivorous mammals. A diet abundant in animal products fosters the proliferation and activity of *Bilophila wadsworthia*, establishing a connection between dietary fat, bile acids, and the cultivation of microorganisms capable of triggering inflammatory bowel disease [54]. These consolidated findings indicate that the gut microbiome undergoes swift changes induced by diet, aiming to facilitate an increase in microbial biodiversity. However, this phenomenon may present a dual aspect, as heightened diversity could also imply an elevation in pathogens at this microbial level.

Dietary changes can rapidly alter the gut microbiome, but the enterotype and other key bacterial community traits remained consistent after a 10-day dietary intervention [54,55].

**Figure 3:**
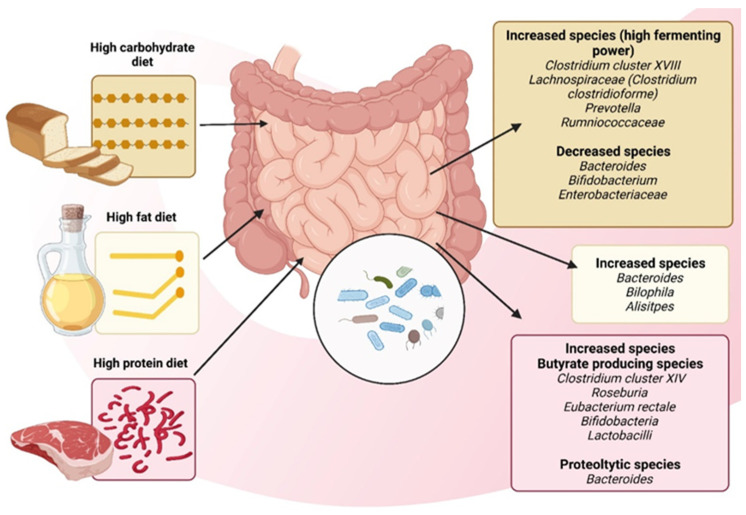
The main bacterial species involved in metabolic processes depending on the major macronutrients from food. Legend: microbiome diversity correlated with foods and nutrition. Created with BioRender.com (accessed on 15 November 2023).

The intricate interplay between dietary patterns and the constitution of bacterial communities within the gut microbiome is undeniable. What we choose to consume profoundly shapes the diversity and abundance of microorganisms residing in our gastrointestinal tract. The prevalence of a specific macronutrient in an individual’s diet can lead to significant modifications in the microbiota configuration, as the microbiome adapts to the digestive processes associated with the predominant macronutrient (see Figure 3). At the same time, certain populations of bacteria, as well as their specific functions, are stimulated as an adaptive mechanism [56].

Natural fibers are a category of carbohydrates from the constitution of plants that are not completely digested in the human intestine. Fibers belong to the category of complex carbohydrates, which, unlike simple ones, are more difficult to digest and absorb in the body, contributing to the establishment of a prolonged feeling of satiety and the inhibition of appetite [57,58].

Although the term ‘dietary fiber’ was introduced in 1953, the health advantages of high-fiber foods have been long recognized. The laxative effects of whole wheat and refined wheat were described by Hippocrates in 430 BC. In the 1920s, J.H. Kellogg has published extensively on the benefits of bran, claiming an increase in stool mass/volume, a laxative effect, and prevention of non-communicable diseases. Dietary fiber was researched throughout the 1930s and then forgotten until the 1970s [57,58,59].

Denis Burkitt (1973) is usually credited with re-popularizing the idea that dietary fiber protects against the development of Western diseases, including diabetes, cardiovascular disease, colon cancer, and obesity [57]. Since then, research has continued to define and understand fiber, analyze it, and determine the health benefits of dietary fiber consumption. Dietary fiber is included in the Nutrition Facts Panels (NFP), and the recommended amount for a food to be considered a good source of fiber is 2.5 g of fiber, respectively 5 g of fiber for an “excellent” source [57,58,59]. The optimal daily fiber intake is subject to variability influenced by age, gender, and individual health circumstances. Commonly recommended by health authorities, the suggested range for adults typically falls between 25 to 35 g per day [59]. Achieving this recommended amount involves incorporating a diverse array of dietary sources, such as fruits, vegetables, whole grains, legumes, nuts, and seeds. Incrementally augmenting fiber consumption and maintaining adequate hydration are essential strategies to avert potential digestive issues.

The interaction between diet and intestinal microflora works in both directions, as gut bacteria can also impact our food preferences by communicating with the brain through the substances they generate [60,61].

Research in recent years regarding the relationship between microbiota and nutrition shows that “we are what our microbes eat” [60,61,62]. The microbiome can be directly affected by diet, which is one of the most important factors [60,61,62].

A topic of conversation among experts is how physical activity frequency can affect the gut saprophytic microbiota composition in children and adolescents. Regular physical activity has been found to improve the diversity of microorganisms in the body, particularly an increase in the phylum *Bacillota phylum*, which includes *Clostridiales*, *Roseburia*, *Lachnospiraceae*. The heightened production of short-chain fatty acids induces an upregulation of tight junction proteins in the colonic epithelium. The increase, especially in the production of butyrate, can promote the upregulation of tight junction proteins in the colonic epithelium. Tight junctions are specialized protein complexes between cells of the intestinal epithelium that regulate the passage of molecules and ions through the paracellular pathway. This dual effect serves to preserve the integrity of the intestinal barrier while concurrently mitigating inflammation [63,64].

Clarke SF et al. (2014) [65] explored how exercise and protein intake affected the saprophytic microbiota in the gastrointestinal tract of 40 professional rugby players from an international team. The athletes were found to have a lower inflammatory profile and better metabolic markers when compared to the control subjects according to the results. Regarding bacterial species, athletes appear to possess superior microbial diversity with over 20 distinct phyla observed compared to controls. Certainly, there is a link between regular or performance-level physical activity and protein consumption, correlating with a higher gut microbiota biodiversity. The results irrevocably prove the beneficial effect of physical activity on saprophytic intestinal microorganisms [65]. The enhanced microbial diversity evident in the gut microbiota of athletes compared to non-athletic individuals may potentially stem from multiple facets of athletes’ lifestyles, encompassing dietary patterns, elevated levels of physical exertion, stress mitigation strategies, and varied environmental exposures. Nevertheless, a thorough comprehension of the causative links between exercise regimens, the status of being an athlete, and the intricacies of microbial diversity necessitates further investigation.

Interestingly, several observational studies seem to have found that exposure to pets and sibling interaction early in life may have a protective effect against allergic diseases [66,67,68]. It has been suggested that children who interact with pets and siblings have higher gut microbial biodiversity, which can help prevent atopy [67]. Tun HM et al. (2017) found that newborns exposed to pets both prenatally and postnatally had higher abundances of *Ruminococcus* and *Oscillopria* (studied in vaginal and cesarean births, respectively), microorganisms negatively correlated with obesity infantile and atopy [69]. Studies have linked *Ruminococcus* to various health conditions such as inflammatory bowel diseases (IBD), obesity, metabolic disorders, and even certain neurological conditions and *Oscillospira* with conditions related to metabolic health, including obesity and type 2 diabetes.

After birth, the gut microbiome develops rapidly. *Bifidobacteria*, microorganisms specialized in the metabolism of milk oligosaccharides, stand out as some of the initial colonizers post-partum [6]. The greater the dietary variety and prolonged exposure to the environment in the first year of life (Table 1), the more the complexity of the microbiome will increase [51,52,70,71].

**Figure 4:**
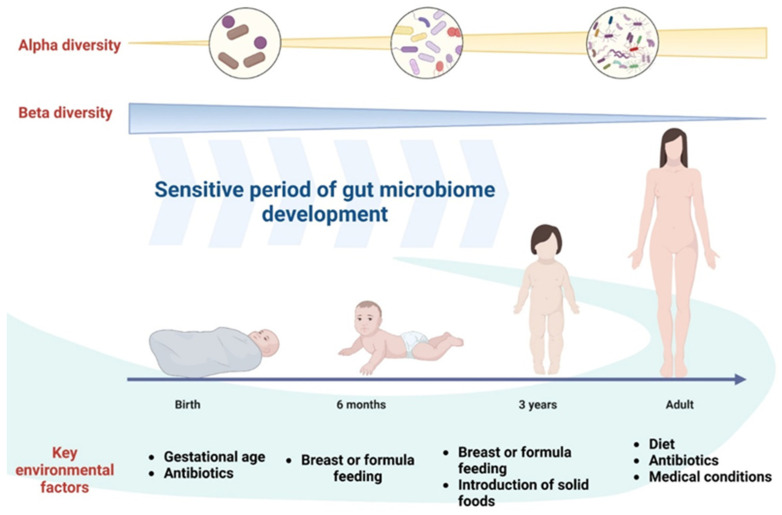
The development process of the intestinal microbiome with aging and key environmental factors. Legend: microbiome diversity correlated with age groups. Created with BioRender.com (accessed on 15 November 2023).

In Figure 4, it can observe how alpha diversity (diversity within a sample) increases as the gut microbiome develops, while beta diversity (diversity between samples) decreases with age, indicating that gut microbiome differences are the most inter-individual variable during childhood and become increasingly similar with adulthood [72].

Alpha diversity in the context of the gut microbiome refers to the quantification of diversity within a specific microbial community residing in an individual’s gastrointestinal tract. This measurement evaluates both the richness, indicating the number of different microbial species or taxa present, and the evenness, reflecting how uniformly these species are distributed within the community. Analyzing alpha diversity aids in understanding the microbial composition and distribution within the gut, offering insights into the health status of the gut ecosystem. Changes in alpha diversity are often associated with various health conditions or dietary patterns, with higher diversity generally linked to improved gut health and potential resilience against certain diseases.

Beta diversity, concerning the gut microbiome, signifies the assessment of diversity discrepancies among different microbial communities rather than within a single community. It encompasses the comparison of microbial composition, structure, or abundance across multiple samples, aiming to discern variations or similarities between these communities. Beta-diversity analysis allows the investigation of how factors such as age, diet, geographical location, health status, and environmental influences contribute to the variability in microbial communities among individuals.

Across age groups, the gut microbial community exhibits numerous similarities as well as distinctions. In individuals over 80 years old, substantial shifts occur in the composition, function, and resilience of the intestinal microbiome. These alterations attributed to senescence seem to stem from imbalances within the microbiome, varying among individuals concerning saprophytic microorganisms and dietary influences. These shifts also align with health markers like indicators of frailty and inflammation [6,73,74,75,76,77,78,79].

### 3.2. Disruptions in Gut Microbiota and Their Impact on Health

Dysbiosis represents an imbalance in the intestinal microbial community that is associated with the presence or development over time of some chronic diseases, administration of broad-spectrum antibiotics, certain medical treatments, and interventions, such as chemotherapy, radiation therapy, and surgeries involving the gastrointestinal tract, a diet characterized by high processed food intake, low fiber content, and limited diversity can influence the gut microbiota, promoting a decline in beneficial microbes while fostering the proliferation of potentially detrimental species, sedentary behavior, poor sleep patterns, excessive alcohol consumption, chronic stress or exposure to environmental toxins and pollutants. This imbalance could be determined by the appearance or loss of some constituent members of the bacterial community, disturbances in metabolic activities, or changes in the relative composition of the number of beneficial microorganisms [72,80,81,82,83]. In general, dysbiosis can be classified into three different types as follows: (i) loss or decrease in beneficial microorganisms, (ii) overgrowth of potentially harmful microorganisms, (iii) loss of overall microbial diversity. It appears that these types of changes can occur simultaneously and not exclusively, so a combination of them is most commonly involved when discussing gut microbiota imbalances [72,81]. Certainly, within the context of the gut microbiota, various factors can disturb the equilibrium and functioning of microbial communities. Among these, interactions and communication between different microbial members play a pivotal role.

At times, even among microbes belonging to the same species, limitations may exist in their ability to effectively communicate or execute specific signaling and metabolic functions. This constraint can stem from genetic variations, differential gene expression, or the presence of distinct strains or subtypes within a given microbial species.

Imbalances in the gastrointestinal tract, known as dysbiosis, have been linked to a myriad of pathologies. These include obesity, malnutrition, type 2 diabetes, hepatic steatosis, chronic hepatitis, metabolic syndrome, irritable bowel syndrome, cardiovascular diseases (hypertension, hypercholesterolemia, atherosclerosis), autoimmune diseases, neurological disorders, neuropsychiatric conditions, neurodegenerative diseases, kidney diseases, respiratory disorders, sarcopenia, cancer, autism spectrum disorders (such as autism and Asperger’s syndrome), sleep disorders, insomnia, allergies, infections (including *Clostridium* difficile infection/CDI), and an exacerbated inflammatory response [6,9,56,82,83,84,85,86,87,88,89,90,91,92,93,94,95,96,97,98,99,100,101,102,103,104]. These diseases are increasingly prevalent in many regions of the world and, to sound the alarm, thirty to forty percent of the world’s population is now affected by one or more allergic diseases, whereas in the past, only a few percent accounted for the total of these diseases [72,105,106,107,108].

Gou W et al. (2020) [109] showed in their study that *Blautia* genus, *Lactobacillus* genus, and *Ruminococcus* genus of *Bacillota phylum* correlate with the severity of the COVID-19 infection. This aspect was also studied by Cai C et al. (2021) [110] who obtained a similar result in colorectal cancer patients who tested positive for COVID-19, and the abundance of these bacteria appears to correlate with worse prognosis in these patients.

Numerous risk factors predispose to gut microbiota imbalance, two of which have been discussed previously (advanced age and a Western diet high in animal fats and processed foods).

As previously pointed out, dysbiosis is sometimes characterized by the excessive growth of harmful/pathogenic microorganisms that generate structural and functional changes at the intestinal level. Certain pathogens, once they reach the enteric level, are able to actively trigger inflammation in the host organism and promote pathogen invasion and dissemination within the host organism. *Campylobacter rodentium*, *Campylobacter jejuni* and *Salmonella enterica serovar*, *Typhimurium* (STm) produce inflammation as part of the infectious process, and an inflammatory process at the intestinal level generates and maintains a disease state [111,112,113,114,115]. The reports indexed in the specialized literature attest and demonstrate that this pro-inflammatory state induced by pathogenic microorganisms affects the intestinal saprophytic microbiota by reducing the number of beneficial bacteria that protect us from infections [115,116,117].

Frank DN et al. (2007) [31] observed that individuals suffering from inflammatory bowel disease (IBD) had smaller numbers of *Lachnospiraceae* and *Bacteroidetes* bacterial populations and *Proteobacteria* families than those in control groups. The development and maintenance of the pro-inflammatory state in the gut have been linked to three major pathogens, as evidenced by studies. These are *Mycobacterium avium paratuberculosis* (MAP), a pathogenic microorganism that was previously associated with the pathogenesis of Crohn’s disease (CRD); Adherent-Invasive *Escherichia coli* (AIEC) frequently found in patients with acute/active IBD and has been correlated with an increased inflammatory response; *Clostridium difficile*, microorganism detected in patients with ulcerative colitis (UC) in relapse and remission [85,86].

In individuals affected by IBD, specific variations in microbial abundance have been observed within certain bacterial genera. Elevated levels of certain species within the *Fusobacterium* genus have been detected in individuals with IBD, indicating a potential association with the condition. Similarly, alterations in the abundance of select species within the *Ruminococcus* genus have been noted in individuals affected by IBD. Although not all *Ruminococcus* species necessarily exhibit detrimental effects, changes in their prevalence have been observed in connection with IBD. Additionally, distinct strains or species within the *Enterococcus* genus have shown increased prevalence or modified abundance in individuals diagnosed with IBD [118,119,120,121]. These findings suggest potential relevance in understanding the microbial signatures associated with IBD, although the specific roles of these microbial species in the pathology of IBD require further investigation.

Research has shown that there are significant changes in gut microbial composition in patients with celiac disease (CD). Studies have demonstrated that untreated CD patients have lower proportions of beneficial bacterial species such as *Bifidobacterium* spp., *Bifidobacterium longum*, *Clostridium histolyticum*, *Clostridium lituseburense*, and *Fecalibacterium prausnitzii* compared to healthy control subjects [119,120,121,122]. Moreover, CD patients have been discovered to have a greater quantity of harmful bacterial species like *Bacteroides* and *Escherichia* than control individuals [121].

Research has verified a decrease in *Fecalibacterium prausnitzii* among CRD patients and showcased that giving *F. prausnitzii* orally reduces colitis severity, displaying potential in addressing dysbiosis. These findings indicate a hopeful avenue in CRD treatment strategy by employing *Fecalibacterium prausnitzii* as a probiotic to restore the disrupted microbiota [118].

In colorectal cancer (CRC), gut microbiota changes were detected with an abundance of *Bacteroides fragilis*, *Enterococcus*, *Escherichia/Shigella*, *Klebsiella*, *Streptococcus* and *Peptostreptococcus* and not a low level in *Roseburia* and other butyrate-producing bacteria belonging to the *Lachnospiraceae* family [123]. Colorectal cancer and *Fusobacterium* spp. Have been linked through genomic analysis [124]. The microbiota of CRC patients is abundant in *Fusobacterium* spp., while (phyla) *Bacteroidetes* and *Bacillota phylum* are found at a very low level. *Fusobacterium* spp. (*Fusobacterium nucleatum* subsp. *Animalis*) appear to contribute to tumorigenesis through an inflammatory mechanism [124].

Antibiotics are the most prescribed drugs, with considerable health benefits. However, antibiotic abuse has been positively correlated with the alteration of the gut saprophytic microbiota and the generation of a disease state with negative impact both in the short and long term [72].

The use of antibiotics often results in the occurrence of *Clostridium difficile* infection (CDI). Individuals across all age groups are affected by it and it is responsible for 15–25% of antibiotic-associated diarrhea. Furthermore, it is the primary cause of colitis associated with antibiotic usage [125].

Treatment with antibiotics causes the decrease in some groups of beneficial microorganisms, causing the important change in metabolism, the increase in intestinal sensitivity to colonization and stimulates the development of bacterial resistance to the antibiotic [126].

Administration of antibiotics during pregnancy is observed to potentially impact the composition and maturation of the intestinal microbiota in the developing fetus. Antibiotics, when taken by pregnant individuals, have the ability to traverse the placenta and reach the fetal environment. This exposure during pregnancy may influence the initial colonization and development of the fetal gut microbiota, a process that begins before birth and continues postnatally. The resulting alterations in the fetal gut microbiota due to antibiotic exposure in utero could potentially affect the establishment of a balanced and healthy microbial community in the infant following birth. These modifications in early gut colonization might have implications for the long-term development of immune function, metabolic processes, and overall health in the offspring. However, further investigation is essential to elucidate the precise impact, timing, and specific consequences of antibiotic exposure during pregnancy on the fetal gut microbiota and subsequent health outcomes in the child. Β-lactam antibiotics, sulfonamides/trimethoprim, macrolides/lincosamides/streptogramins are commonly prescribed during pregnancy to combat urinary tract infections, respiratory infections, skin or ear infections, bacterial vaginosis, and fever of unknown origin [127,128]. Mothers are commonly given antibiotics during labor as a preventive measure against the transmission of group B *Streptococcus*, to lower the risk of endometrial infection, and to prevent wound infections [129]. Nevertheless, the World Health Organization (WHO) advises against the prophylactic use of antibiotics in uncomplicated labor. This caution arises from evidence demonstrating that the exposure of newborns to antibiotics through intrapartum antibiotic prophylaxis (IAP) can lead to alterations in gut microbial diversity [130].

**Figure 5:**
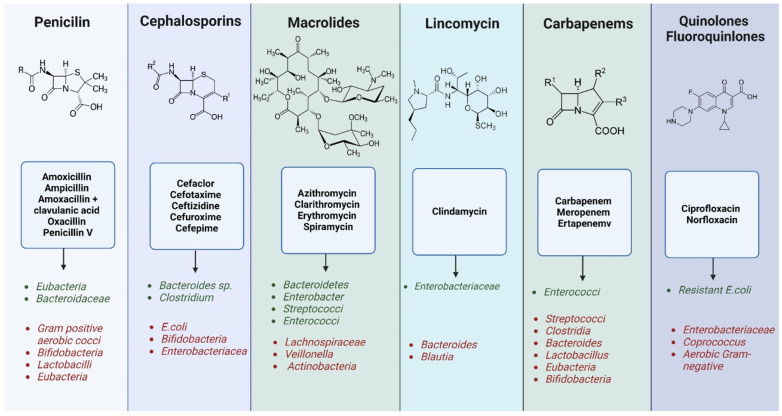
Antibiotic classes and bacteria affected by their administration. Legend: microbiome diversity correlated with antibiotic classes. Created with BioRender.com (accessed on 15 November 2023).

Studies on healthy adults have revealed the long-term consequences of antibiotics like amoxicillin, ciprofloxacin, and cefrozil. These studies reveal persistent alterations in microbial biodiversity for up to 12 weeks post-treatment (Figure 5). Furthermore, there’s evidence indicating incomplete restoration of microbial composition and the emergence of antibiotic-resistant strains [131,132,133]. Another study investigated the gut microbiota of subjects over a 10-month period following administration of the antibiotic ciprofloxacin. The results showed that the effects of ciprofloxacin were rapid and significant on microbial biodiversity with a decrease in beneficial bacteria and changes in concentrations of *Bacteroidetes*, *Lachnospiraceae* and *Ruminococcaceae*. One week after each analysis, microbial communities began to return to baseline, but incompletely and variably compared to baseline [134].

Short-term use of clindamycin over 7 days resulted in significant changes in bacterial communities with a sharp decrease in *Bacteroides* and *Enterococcal* colonies (red labels), and these changes persisted up to 2 years post-treatment and were accompanied by increases in of antibiotic-resistant genes and strains (green labels) [135,136,137].

Given the fundamental nature of microbial life and diversity relative to larger organisms and vice versa, scientists are calling for a reconsideration of the role of microorganisms [138,139,140]. Despite the substantial popularity of microbiome research in various fields, this discipline of extremely rapidly growing interest faces a variety of challenges. The lack of data standardization is caused by the continuous development of new techniques and equipment, as well as the urgent need for better coordination and collaboration in the field of microbiome research [141].

The saprophytic microbiota at the intestinal level has many important functions in the body (Table 1), so maintaining its composition in optimal conditions is one of the foundations of a healthy and balanced lifestyle.

**Table 1:** Key roles of intestinal microbiota in human health (implications on its metabolism, immunity and trophicity).

Key Roles of Intestinal Microbiota in Human Health [142]		
Metabolic	Immunity	Trophicity
The production of short-chain fatty acids, including acetate, butyrate, and propionate, contributes to several beneficial effects on the host organism Vitamin synthesis (vitamins K, B12) [143,144] Increases absorption of calcium, iron and magnesium [145,146,147] Participates in metabolism fatty acids [142] Participates in the degradation of polyphenols [142] Participates in the degradation of choline and amino acids [142] Participates in the production of polyamines [142] Metabolism of xenobiotics (drugs) [142]	Interacts with pathogens for nutrients and attachment sites (receptors) [142] Secretion of antimicrobial components (bacteriocins, lactates) [142] It induces the synthesis of antimicrobial proteins (cathelicidins, C-type lectins, defensins) [142] Stimulates the production of IgA immunoglobulins [142,148] Regulates the development and functioning of the immune system [142]	Regulates intestinal epithelial development through cell proliferation and differentiation (angiogenesis, crypt formation) [149] Stimulates intestinal peristalsis [142] Effects on systems and viscera (central nervous system, liver, heart, lungs) [142] Butyrate resulting from fermentation has an inhibitory effect on neoplastic cells [150]

## 4. The Relationship of the Microbiome with Different Parts of the Body

### 4.1. The Microbiota–Gut–Brain Axis

The gut–brain axis is a complex communication network between the gut, microbiota, and brain. This axis involves a multitude of physiological, biochemical, and neural pathways that facilitate communication between these three major components. This intricate connection is governed by the nervous, immune, and endocrine systems, facilitating a bidirectional communication system [151,152,153]. Direct neurochemical signals are received by both the brain and gut microbiota through the vagus nerve, which creates a vital link between the enteric nervous system and the central nervous system (CNS). This connection is a significant element of the microbiota–gut–brain axis [154,155]. The vagus nerve plays a central role in facilitating direct neurochemical communication between the brain and the gut microbiota, establishing a bidirectional link between the enteric nervous system (ENS) within the gut and the central nervous system (CNS). This intricate pathway forms the foundation of the microbiota–gut–brain axis. Through the vagus nerve, various signaling molecules, encompassing neurotransmitters, neuropeptides, and other bioactive compounds, are exchanged between the gut microbiota and the brain. This reciprocal communication allows signals from the gut microbes to influence neural processes in the brain and, conversely, permits brain-derived signals to modulate activities within the gut.

This neural pathway significantly regulates a spectrum of physiological functions, spanning gastrointestinal activities, immune responses, stress reactivity, mood regulation, and cognitive functions. The interplay between the gut microbiota and the CNS through the vagus nerve constitutes a critical component of the microbiota–gut–brain axis, profoundly impacting how gut microbes influence brain health and behavior.

The enteric nervous system (ENS), also called the second brain, houses a nerve count equivalent to that of the spinal cord. It oversees intestinal peristalsis, controls intestinal permeability, regulates enteroendocrine signaling, and orchestrates mucosal immune activity, exhibiting neurotransmitters and signaling molecules akin to the brain.

Multiple paths facilitate communication between the brain and the digestive tract. These pathways include the hypothalamic-pituitary-adrenal (HPA) axis, the sympathetic and parasympathetic divisions of the autonomic nervous system (ANS), and the sympatho–adrenergic axis. The sympatho–adrenergic axis plays a role in modulating gut microbiota-associated lymphoid tissue [90]. Vagal afferents, comprising 80–90% of total vagal fibers, predominantly transmit signals from the gut to the brain. These afferents also interact with the HPA axis, coordinating stress responses and anti-inflammatory actions [156].

The gut microbiota is recognized as the largest endocrine organ within the human body, generating a diverse array of hormones and bioactive peptides [157]. Gut–brain communication is facilitated by extremely chemo-sensitive primary afferent neurons, housing more than 30 distinct hormones. The communication between the gut and the brain relies on highly sensitive primary afferent neurons adept at detecting and responding to chemical signals. These neurons act as conduits, relaying information bidirectionally from the gut to the brain and back. They house a diverse array of more than 30 distinct hormones. These primary afferent neurons possess specialized receptors that perceive a range of chemical cues present within the gut milieu, including signals originating from gut microbes, nutrients derived from ingested food, and endogenous biochemical substances produced within the gastrointestinal tract. Upon sensing these chemical stimuli, these neurons transmit this information to the brain through neural pathways, such as the vagus nerve or spinal cord, establishing the gut–brain axis [157]. Additionally, signals originating from the brain can influence the activity of these neurons, regulating various aspects of gut function and responses. The multitude of more than 30 hormones harbored within these neurons function as signaling molecules capable of modulating diverse physiological processes. These functions include regulating gut motility, orchestrating the release of digestive enzymes, governing appetite, and influencing mood. These hormones contribute significantly to the intricate network facilitating communication between the gut and the brain, playing integral roles in regulating numerous bodily functions and behaviors. Grasping the complexities of this communication system is essential for comprehending the bidirectional interactions between the gut and the brain. Disruptions or dysregulation in this gut–brain communication network can have implications for various health conditions, underscoring the significance of this neural network in maintaining overall health and homeostasis.

Additionally, from an immune system perspective, cytokine signaling triggered by microbial components like lipopolysaccharides (LPS) or peptidoglycans serves as a communication route to the brain. Structural changes in the intestinal barrier predispose to a re-localization of these microbial products to the periphery which causes downstream microglial activation and neuroinflammation [158].

When there is a disruption in the natural balance of microorganisms in our intestines, known as dysbiosis, it can negatively affect the relationship between the gut and the brain. The gut microbiota and its communication with the central nervous system are both affected by various diseases, making this complex system susceptible to influence from various illnesses. The microbial composition, diversity, and functionality of the gut can be altered by conditions such as inflammatory bowel disease (IBD), irritable bowel syndrome (IBS), gastrointestinal infections, and metabolic disorders, resulting in a state of dysbiosis. Simultaneously, diseases affecting the CNS, including neurological disorders like Parkinson’s disease, multiple sclerosis, and mood disorders, can disrupt neural signaling pathways involved in gut–brain communication. This bidirectional relationship underscores the susceptibility of the gut microbiota–CNS axis to influence from diverse illnesses. Perturbations caused by these conditions can potentially lead to dysregulated gut–brain communication, emphasizing the need for a comprehensive understanding of these interactions in the management and treatment of associated health conditions. Disruption in the microbiota–gut–brain axis results in heightened oxidative stress and inflammation, along with disturbances in metabolic and immune functions [159].

Multiple studies attest to the ability of microbes to produce neurotransmitters (like gamma-aminobutyric acid, dopamine, and serotonin), promote serotonin production by intestinal epithelial cells, produce bioactive constituents (short-chain fatty acids), alter epigenetic regulation with fermentation products (can alter gene expression patterns without changing the underlying DNA sequence, affecting various cellular processes and potentially impacting health outcomes), and release metabolites that can enter the general circulation and cross the barrier hemato-encephalic [160]. Elderly patients as well as those affected by a neurodegenerative pathology present a reduced intestinal microbial biodiversity [79,161,162].

Dysbiosis and reduction in microbial biodiversity observed as changes associated with senescence and neurological processes contribute to the neuroinflammation underlying neurodegenerative diseases. Gut microbiota contributes to neuroinflammation through various pathways, such as via endotoxic constituents in the bacterial cell wall (e.g., LPS or microbiota-produced amyloid). Increasing the abundance of butyrate-producing bacteria can ameliorate neuroimmune activation.

In instances of neurodegenerative conditions such as Parkinson’s and Alzheimer’s diseases, a phenomenon occurs involving the misfolding of pivotal proteins, resulting in their clustering and buildup within the brain. The proteins associated with the degenerative processes are alpha-synuclein in the case of Parkinson’s disease, and beta-amyloid as well as tau protein in the context of Alzheimer’s disease [153,163,164].

The intestinal microbiota, or more precisely, its imbalance, leads or maintains certain specific neurological, neuropsychiatric, and neurodegenerative pathologies, such as Parkinson’s disease, Alzheimer’s disease, depression, anxiety, schizophrenia, dementia, anorexia nervosa (AN), autistic spectrum disorders (autism, Asperger’s syndrome) and sleep disorders and insomnia [6,9,56,89,90,95,102]. Individuals experiencing mood disorders often exhibit variations in the diversity and quantities of specific microbial species compared to those without these conditions. These alterations may involve decreases in beneficial bacteria like *Lactobacillus* and *Bifidobacterium*, alongside shifts in other microbial groups. Imbalances in the gut microbiota, termed dysbiosis, might contribute to heightened gut permeability and an inflammatory environment [89,95]. This disturbed gut milieu could trigger the production of inflammatory molecules that may impact pathways linked to mood regulation in the brain. Gut microbes are involved in synthesizing neurotransmitters such as serotonin, dopamine, and gamma-aminobutyric acid [90,102]. Changes in microbial populations might affect the production of these neurotransmitters, known for their roles in regulating mood. For instance, reduced gut serotonin levels might influence mood disorders due to serotonin’s involvement in emotional well-being. The bidirectional communication between the gut and the central nervous system, known as the gut–brain axis, is modulated by the gut microbiota. Disruptions in this communication may contribute to alterations in mood regulation and cognitive processes.

However, the relationship between mood disorders and the gut microbiota is intricate and influenced by various factors such as diet, lifestyle, medications, and individual differences. Ongoing research aims to elucidate the precise mechanisms underlying these associations and explore potential therapeutic interventions targeting the gut microbiota for managing mood disorders.

### 4.2. The Relationship of the Gut Microbiome to the Cardiovascular System

Saprophytic bacteria in the gut can generate metabolic products derived from the diet that can influence the state of the host’s cardiovascular system. As an illustration, elevated levels of amino acid metabolites like tryptophan and histidine have been linked to heightened insulin resistance and the onset of cardiovascular disease [165]. Another notable instance is imidazole propionate, a byproduct generated through histidine metabolism. In individuals with both obesity and diabetes, there seems to be a significant increase in the concentration of imidazole propionate in the portal vein, as opposed to individuals with obesity alone and without diabetes [166].

As previously talked about, the gut microbiome can communicate with various organs, including the heart, through different pathways. These pathways include compounds such as trimethylamine (TMA)/trimethylamine N-oxide (TMAO), short-chain fatty acids (SCFA), bile acids, lipopolysaccharide (LPS), and peptidoglycan [166]. TMAO, which is a well-researched metabolite produced by the local saprophytic microbiota in the gut, is associated with atherosclerosis. TMA is formed by the intestinal microbiota after consuming meals that are rich in choline, phosphatidylcholine, or carnitine, which are commonly found in foods with higher saturated and unsaturated fat content. Since the human body does not possess TMA-lyase, the intestinal microbiota is responsible for producing all TMA. After being absorbed, TMA travels to the liver, where the liver enzyme flavin monooxygenase-3 (FMO3) oxidizes it to TMAO [165].

Elevated serum TMAO levels are positively associated with early atherosclerosis, and monitoring these levels aids in predicting mortality risk among patients with stable coronary artery disease or acute coronary syndrome [167,168]. Increased plasma TMAO levels are also linked with the severity of peripheral arterial disease and an elevated risk of cardiovascular death in individuals diagnosed with peripheral arterial disease [169].

Dose–response meta-analyses have shown a correlation between elevated plasma TMAO levels and a higher incidence of major cardiovascular events in coronary artery disease patients [170]. Furthermore, heightened TMAO levels have been associated with pro-inflammatory monocytes in stroke patients [170]. Haghikia A et al. (2018) reported that increased TMAO levels were linked to a higher predisposition to cardiovascular events like myocardial infarction, recurrent stroke, and cardiovascular death [171].

Not all products of food fermentation are harmful. Some can actually benefit our cardiovascular health. Emerging evidence suggests that short-chain fatty acids (SCFA) play a crucial role in regulating the inflammatory response of intimal atheroma plaque, a condition that is often associated with heart disease. Studies have demonstrated that SCFAs activate free fatty acid (FFA) receptors and G-coupled proteins, while also inhibiting histone deacetylases (HDACs) to promote a more favorable inflammatory response [172]. This mechanism of action has been found to have a positive impact on the progression of atheroma plaque, making SCFAs a promising avenue for the prevention and treatment of heart disease.

Butyrate, a short-chain fatty acid, is known to impede the progression of atherosclerosis, while propionate inhibits cholesterol synthesis and its accumulation in the liver [5,59].

Bile acids also have a protective effect on cardiovascular disease (along with antioxidants, fibers, polyunsaturated fatty acids). They have an interesting route starting from the liver after the transformation of cholesterol into primary bile acids (cholic acid and chenodeoxycholic acid), they combine with taurine (taurocholic acid and taurochenodeoxycholic acid) and glycochol (glycocholic acid and glycochenodeoxycholic acid), and then are discharged with bile in the duodenum, reducing the acidity of the pH and improving the solubility of many nutrients [165,173]. In addition to this absorption effect of lipids and fat-soluble vitamins, bile acids are involved in metabolic processes, intestinal motility, inflammatory processes, as well as liver regeneration. Bile acids exert these effects via bile acid receptors with farnesoid X receptor and TGR5 receptor as major contributors, present in numerous cells and tissues. Bile acid binding to FXR lowers lipid levels, improves insulin sensitivity, and inhibits hepatic gluconeogenesis, while stimulation of the TGR5 receptor reduces cytokine production [174].

Other mechanisms suggested by the specialized scientific literature for the cholesterol-reducing effects are inhibition of the hepatic synthesis of fatty acids by the products of intestinal fermentation of fibers (short-chain fatty acids: butyrate, propionate); the mechanism of intestinal motility change; the delay in the absorption of macronutrients and the faster and longer-term establishment of the feeling of satiety. A long-lasting feeling of satiety causes a decrease in caloric intake, implicitly also in fatty acids from food [5,59,61,175,176,177,178,179,180].

Diets high in soluble fiber significantly lower total cholesterol and LDL-cholesterol, but also appear to have the undesirable effect of lowering HDL-cholesterol. However, the impact on HDL cholesterol is not obvious, being at the limit of statistical significance [176,177].

The gut microbiome is pivotal in preserving the equilibrium of the cardiovascular system or, conversely, in fostering and sustaining disease conditions. Mounting evidence supports the notion that an active lifestyle, coupled with a well-rounded diet, consistent physical exercise, and cessation of smoking, confers a protective impact on the intestinal microbiota. By extension, this care for the gut microbiota positively influences the cardiovascular system. Health professionals increasingly advocate for these practices in promoting cardiovascular wellness [181]. Intestinal microbiota can represent, by altering the connections with the heart, a risk factor for the onset of myocardial infarction, heart failure, atherosclerosis, hypertension, myocardial fibrosis and coronary heart disease [101].

### 4.3. The Gut–Muscle Axis

Micronutrients and metabolites derived from the metabolic processes of the intestinal microbiota can reach and even act on the muscles [182]. Therefore, the concept of the gut–muscle axis has emerged to study this link [183]. The advancement of research and specialized literature in this sense has led to the conclusion of a possibility that through this axis, reversible effects on sarcopenic phenotypes can be exerted [182]. *Lactobacillus* and *Bifidobacterium* supplementation appears to have a significant role in increasing muscle mass, muscle strength, and endurance in aged rats [184]. Scientific investigations have demonstrated that older individuals can derive advantages from these positive impacts. Consequently, interventions targeting the gut–muscle axis possess the potential to postpone the onset of muscle deterioration and age-related dysfunction (sarcopenia) [183].

Ni Y et al. (2019) conducted an investigation on the administration of *Lactobacillus casei* LC122 and/or *Bifidobacterium longum* BL986 in aged mice for their anti-aging and other beneficial effects [184]. Aging led to a reduction in muscle weight, including the quadriceps, gastrocnemius, and tibialis muscles. Administration of *Lactobacillus* significantly increased gastrocnemius muscle weight, while *Bifidobacterium* treatment significantly increased both gastrocnemius and tibialis muscle weights. Soleus muscle weight was unaffected by aging or probiotic treatment. Grip strength, an indicator of muscle function, decreased with age but improved with probiotics. Probiotics also enhanced physical endurance, as shown by reduced immobility time in a forced swimming test (FST), and upregulated genes related to muscle survival, differentiation, and hypertrophy (salt inducible kinase 1, peroxisome proliferator-activated receptor gamma coactivator 1-alpha 4) [184]. Several mechanisms may explain the improvement of muscle mass and function upon administration of probiotics. It was suggested that probiotics, like *Lactobacillus* and *Bifidobacterium*, may have beneficial effects on muscle anabolism, therefore causing reduced inflammation, and also may improve gut barrier function and decrease systemic inflammation [185]. Firstly, these environments might be conducive to muscle protein synthesis compared to breakdown. Secondly, the expression of genes leading to muscle growth and endurance might be influenced by probiotics either directly at the gene level or indirectly, through modulation of the signaling pathways involved in muscle metabolism and regeneration. More research is necessary to explain the exact molecular mechanism of how probiotics act toward the beneficial effect on muscle health.

A systematic review and meta-analysis of 24 randomized clinical trials (RCTs) proposed some mechanisms regarding the influence of probiotics on optimizing and enhancing muscle function [185]. Notable is the role played by the amino acids, particularly post-prandial leucine, in upregulating muscle protein synthesis (MPS), even further boosted by resistance exercise. Probiotics such as *Bacillus coagulans* GBI-30, 6086 (BC30) enhanced protein digestion and showed increased amino acid blood levels in human trials (arginine, isoleucine, serine, methionine, glutamic acid, tyrosine, phenylalanine, and total amino acids). Also, it has been observed that SCFAs produced by gut bacteria reverse the impairments in muscles in mouse models, and with increasing age, older adults who exhibited higher levels of butyrate also demonstrated greater lean mass. This difference in creatine’s degradation by the microbiomes between older and younger mice further drives the gut’s support in muscle health, perhaps hinting at a possible combating role against sarcopenia [185].

As in any domain, despite the significant advancements achieved through increased research into the intestinal microbiota and its connection to various niches of the human body, scientists acknowledge that there remain unanswered questions and areas unexplored in scientific studies. Regarding the microbiota-muscle axis, there are uncertainties about the clear mechanisms by which the microbiota acts and influences the physiology of the muscular system. Furthermore, most of the evidence presented supporting the gut–muscle axis is from animal models. Therefore, many human-based studies need to be considered in validating such findings and proving their importance in human health and disease [186]. While short-term studies have provided initial insights, there’s a pressing need for long-term research to deeply understand how gut microbiota persistently affects muscle health. This extended analysis is essential to ascertain the long-lasting benefits or potential drawbacks of interventions targeting the microbiota, ensuring a comprehensive view of their impact over time.

### 4.4. The Gut–Bone Axis

While many of the studies in the specialized literature discuss changes in the structure of the intestinal microbiota related to obesity, gastrointestinal and cardiometabolic disorders [3,9,187], new genomic investigation methods currently allow the study of intestinal microbial interactions with peripheral tissues, like bone. Moreover, several mechanisms have been analyzed that attest to the existence of the gut–bone axis, through which intestinal saprophytic microorganisms play an important role in health [188].

This concept significantly expands our understanding of the intestinal microbiota’s impact on skeletal system health, going beyond its conventional role of facilitating mineral absorption, which is essential for proper functioning [189].

A growing number of studies in the literature show that prebiotics are essential for improving the intestinal absorption of calcium and other minerals and also for improving the health of the musculoskeletal system [190,191].

Many clinical trials involving prebiotics have helped to better understand the potential mechanisms by which the microbiota influence bone health, but gaps remain regarding the mechanism by which prebiotics act (directly or indirectly) through the gut–bone axis to prevent age-related bone loss and osteoporosis.

A study that analyzed the effectiveness of a mix between galacto-oligosaccharides and fructoligosaccharides (GOS-FOS 5.3%) over 50 days on *Wistar* rats found a significantly higher concentration of calcium and phosphorus at the femoral level. At the same time, the association between GOS and FOS (GOS+FOS) resulted in a greater length of the tibia compared to other diets, having a potential role in the bone growth process. *Lactobacillus* colonies were also increased in the GOS+FOS group [192].

There are multiple biochemical, physiological, immunological, cellular, and molecular mechanisms involved in preventing bone loss and ultimately optimizing bone metabolism. It appears that reduced levels of TRAP 5 glycoprotein and RANKL transmembrane molecule are correlated with the prevention of bone loss. Other mechanisms involve inhibition of T-cell signaling activity, inhibition of basal tumor necrosis factor (TNF) mRNA production, and regulation of Treg-Th17 cell differentiation [193].

The molecular mechanisms described by the authors involve the regulation of bone metabolism and calcium homeostasis through interactions between dietary components, gut microbiota, and their metabolic by-products, such as SCFAs. SCFAs, produced by the fermentation of prebiotics by gut microbiota, lower intestinal pH and interact with proteins to enhance the expression of calcium transporters like calbindin-D9k and TRPV6, facilitating calcium absorption [193].

At the same time, modulation and modification of the intestinal microbiota significantly participate in the process of bone remodeling (balance between the decomposition/resorption of old bones and the creation of new bones, a process governed by osteoblasts and osteoclasts). This regulation is mediated by immune system components, particularly through the activation of T lymphocytes in the gut, which then influence bone marrow activities [194]. The RANK-RANK ligand pathway is pivotal in this process, with cytokines like TNF and various interleukins playing roles in promoting or inhibiting osteoclastogenesis [195]. Additionally, the gut microbiome’s interaction with estrogen levels impacts bone density, with probiotics showing the potential to mitigate bone loss by modulating immune responses and reducing inflammation [196].

In addition to these two mechanisms, calcium-regulating hormones play a crucial role in bone health, with various hormones modulating bone metabolism. It is suggested that the gut microbiome may promote the anabolic processes in bones through the action of insulin-like growth factor (IGF-1), essential for juvenile bone growth through paracrine and endocrine actions [197]. Furthermore, interactions between the microbiota and the host influence IGF-1 production and metabolism. Parathyroid hormone, significant for skeletal muscle development, may be stimulated by microbiota-produced metabolites, suggesting a complex interplay between microbiota and bone formation mechanisms [198].

The vital importance of the gut microbiome in regulating bone health is underscored by the detailed and complex mechanisms involved. This understanding opens up the possibility of developing new therapeutic approaches to treat conditions like osteoporosis by adjusting the gut microbiota through diet.

### 4.5. The Relationship of the Gut Microbiome to the Immune System

The development of the host’s immune system is heavily dependent on microbial colonization of mucosal tissues during childhood. These events that occur early in life can have long-term consequences: improving tolerance to exposure to environmental factors or maintaining a disease state later with age (inflammatory bowel disease, allergy, and asthma) [199].

The interactions between the host immune system and the gut microbiota have been the focus of many studies in the last decade [200]. The innate immune system swiftly reacts to the saprophytic gut microbiota in a manner that is not specific to antigens. It does so by engaging pattern recognition receptors and releasing cytokines like interferon-α, interleukin-18 (IL-18), and IL-22. These facilitate optimal responses in epithelial antimicrobial activities, such as the generation of antimicrobial peptides [201]. Furthermore, newly characterized innate lymphoid cells imitate T-cell cytokine production in a manner that lacks specificity to antigens [202].

Evidence suggests that reduced biodiversity and balance during microbiome development, generally from 1 to 3 years of age, may increase the incidence of allergies and autoimmune diseases. For instance, children delivered via caesarean section displayed a distinct microbial composition compared to those born naturally through the vaginal canal. Additionally, it appears that these children have a heightened likelihood of developing allergies and asthma [203,204].

Alteration of the intestinal microbiota (dysbiosis) has been associated with a variety of immune pathologies such as asthma, allergies, dry eye syndrome, and Sjögren’s syndrome [205].

Table 2 presents a comprehensive overview of microbiota alterations associated with a spectrum of pathologic conditions, encompassing both predisposing factors such as acute or chronic stress and profound diseases with life-threatening implications, including cardiovascular diseases and neurodegenerative disorders. The collected data present the intricate interplay between the composition of the microbiota and diverse pathological states, thereby underscoring the potential significance of the microbiome in the context of health and disease.

The gut microbiota exerts a profound impact on the regulation of both innate and adaptive immune system components, consequently shaping the body’s overarching protective measures against pathogens and facilitating health preservation. This brief overview will shed light on the interactions between the gut microbiome and the immune system, as well as the key molecules involved in the foundational processes of this interaction.

The communication between the gut microbiome and the mucosal immune system plays a pivotal role in safeguarding health, acting as the primary barrier against invasive microbes. This interface is equipped with specialized immune mechanisms, such as a robust mucus layer, tight junction proteins, and antimicrobial agents. Innate immune cells in the intestines learn to tolerate beneficial bacteria while blocking harmful pathogens from entering the bloodstream [251]. When invasive bacteria penetrate the epithelial barrier, they, together with pathogen-associated molecular patterns (PAMPs) such as lipopolysaccharides (LPS), stimulate goblet cells to secrete mucin. PAMPs trigger immune responses by engaging Toll-like receptors (TLRs) on neutrophils and macrophages, essential components of the innate immune system. Beneficial gut bacteria activate dendritic cells (DCs) by presenting antigens, which in turn triggers TLR activation, facilitating immune system priming and response differentiation between harmful and harmless microbes. Beyond macrophages, neutrophils, and dendritic cells, specialized epithelial cells like goblet and Paneth cells also contribute to gut immunity. They secrete antimicrobial substances, including mucins, defensins, lysozyme, secretory phospholipase A2, and cathelicidins, acting as supportive immune elements to maintain the gut’s innate defense mechanisms [251,252].

In clinical illness, changes in the gut’s environment can cause dysbiosis, leading to imbalanced immune responses. This environment causes neutrophils to overreact, potentially causing damage through increased inflammatory signals, enzyme production, and abnormal immune cell activation. Normally, neutrophils are regulated to avoid disrupting the balance of gut bacteria, a process controlled by specific proteins. Interestingly, while the formation of neutrophil extracellular traps (NETs) can help eliminate pathogens and reduce inflammation, antibiotic-induced dysbiosis can lead to NET formation that exacerbates inflammation. This suggests the need for further research on the role of NETs in the gut. Maintaining a balance between the immune system and gut bacteria is crucial for health and preventing disease [251].

The gut mucosa’s adaptive immune system primarily consists of intraepithelial lymphocytes (IELs) and lamina propria lymphocytes (LPLs). Within the IEL group, γδ T cells stand out due to their expression of the Helios transcription factor, marking them as a unique subset of T cells. γδ T lymphocytes halt the spread of bacteria in the gut by secreting protective cytokines and proteins, activating CD4+ T cells for immune responses like IL-22 production. Their growth is supported by specific gut bacteria and metabolites, such as those from Desulfovibrio, showcasing the complex interplay between the microbiota and immune defenses [253].

Preventing bacterial spread and infection is dependent on the vital relationship between gut microbiota and the adaptive immune system. It has been shown that colonization of germ-free mice, whose adaptive immune system is less active, by commensal bacteria would stimulate the development of important mucosal lymphocytes such as CD4+ T cells and cytotoxic CD8+ T cells, underlining the importance of microbiota in immune system maturation and defense mechanisms [251].

Key modulators of these interactions are Th17 and Treg cells which have distinct roles, participating in both body protection and inflammatory responses. T helper 17 cells play a dual role in both defending the body and triggering inflammatory responses. Although Th17 responses are mostly considered harmful, with intestinal Th17 cells fostering disease-causing T cells in diseases outside the intestine, not all Th17 activity is detrimental. Th17 cells activated by segmented filamentous bacteria (SFB) are non-inflammatory, in contrast to those stimulated by *Citrobacter* spp., which are inflammatory. The presence of Th17 cells is dependent on specific microbes like SFB, which are absent in germ-free mice [251].

Regulatory T cells are pivotal in establishing immune tolerance within the gastrointestinal tract. Originating from the thymus for self-tolerance, their peripheral proliferation is further influenced by diet and microbiota interactions. Gut microbiota, through diverse mechanisms, stimulates Treg development, notably with ILCs (Innate lymphoid cells) fostering microbiota-specific RORγt+ Tregs to counterbalance Th17 cell proliferation, ensuring intestinal immune harmony [251,254]. Intriguingly, specific microbes such as *Helicobacter* spp. and *Akkermansia muciniphila* are capable of promoting RORγt+ Treg-mediated responses, highlighting the nuanced interplay between diet, microbiota, and immune tolerance mechanisms [255].

Given the complexity of the interactions between gut bacteria and the individual’s immune system, it’s clear that understanding these mechanisms requires a collaborative effort from experts across different fields. The goal is to pinpoint imbalances that might interfere with these detailed processes. Delving into how gut bacteria affect the development and functionality of the adaptive immune system opens new possibilities for rethinking and redefining treatment strategies for immunological disorders. This exploration could further highlight the symbiotic relationship between commensal bacteria and humans that has evolved over time.

## 5. Principles of Nutritional Therapy for Balancing the Intestinal Microbiome

The protective effect of a balanced and healthy diet on the human body is already well known. Macronutrients and micronutrients that come from food are transformed at the intestinal level by saprophytic bacteria, and the resulting products are either used as an energy substrate or involved in various local and systemic processes. At the same time, a Western-type diet can alter the intestinal microbiota and induce the production of dangerous metabolites that exert negative effects on the body.

Among the foods that improve the health of the body are soluble fibers, which represent a category of carbohydrates from the constitution of plants that are not completely digested in the human intestine, they undergo a fermentation process [57,58]. Although how fiber works in the consumer’s body is not fully understood, nutritionists and health professionals unanimously recognize its therapeutic benefits.

Prebiotics was first defined as “non-digestible food ingredients that beneficially affect the host organism by selectively stimulating the growth and/or activity of one or a limited number of bacteria in the colon, thereby improving the health of the hos”. The revised definition includes more comprehensive language regarding the microbiota, specifically identifying changes throughout the entire gastrointestinal tract rather than just the colon, and bacteria quantity is no longer limited to just one: “Ingredients that through selective fermentation allow changes in composition and the activity of saprophytic flora (probiotics) and stimulates the activity of *Bifidobacteri*”. *Lactobacilli* and *Bifidobacteria* are the usual target species for prebiotics; changes in *Bifidobacteria* are more likely to be observed compared to *Lactobacilli*. This can be explained by the fact that in the human large intestine, we encounter a greater number of *Bifidobacteria*, compared to lactobacilli, and show a preference for oligosaccharides [21].

The classification of a food ingredient as a prebiotic requires its scientific demonstration and the fulfillment of the following three criteria:

(i.) Can be absorbed in the upper gastrointestinal tract without being broken down by gastric acidity or mammalian enzymes;
(ii.) It is fermented by bacterial microflora from intestine;
(iii.) It selectively stimulates the growth and/or activity of saprophytic intestinal bacteria associated with well-being and health [21].

Fermentability, solubility, and viscosity are the physicochemical characteristics of fibers, which affect both fermentation and the therapeutic effects of their consumption. Psyllium may not be a fermentable fiber, but it has unique therapeutic effects, such as improved glucose control and reduced blood cholesterol levels, due to its high solubility and viscosity. β-glucans and pectins stand out as fibers with a combination of high fermentability, solubility, and viscosity. Through intestinal fermentation, soluble dietary fibers with a low degree of viscosity (pectins and fructo-oligosaccharides) promote the production of SCFAs, including acetic, propionic, and butyric acids, as well as carboxylic acids such as lactic acid [5,21,57,58,60,61,256,257,258].

Extensive studies have explored the capacity of diet to modify the gastrointestinal microbiota in humans and other mammals. Research suggests that the gut microbial communities are influenced by both the composition and habitual intake of the diet, as well as acute dietary changes. Numerous studies have demonstrated that diets rich in fiber significantly lower the risk of developing diabetes and obesity [259,260]. The widespread adoption of Western dietary patterns has played a role in diminishing fiber consumption, leading to an increase in health conditions such as cardiovascular disease, obesity, diabetes, and other chronic non-communicable diseases. Physiological studies have shown that fiber can help lower cholesterol levels, increase calcium absorption, boost immunity, and maintain gastrointestinal health, while also regulating blood sugar levels [60,261].

In the category of prebiotics, i.e., fibers that meet the 3 criteria mentioned above, include inulin and oligofructose (OF), lactulose, resistant starch (RS), physically and/or chemically modified starch, galactooligosaccharides (GOS), trans-galactic-oligosaccharides (TOS), polydextrose, hydrocolloids (gums, mucilages, β-glucans), psyllium, pectins, soluble corn fiber and other non-digestible oligosaccharides (raffinose, stachyose, inulin/ITF-type fructans, galactans, and mannans) [57,259,262].

Resistant oligosaccharides, alternatively known as functional oligosaccharides, are oligosaccharides characterized by a degree of polymerization (DP) ranging from 3 to 9. These particular oligosaccharides exhibit prebiotic effects, promoting the growth and activity of beneficial microorganisms in the gut. Due to their low molecular weight and high solubility, fructo-oligosaccharides (FOS) and galacto-oligosaccharides (GOS) are readily fermented in the intestine [57,58,256].

Food sources of prebiotics include soybeans and other legumes (chickpeas, beans, lentils, peas), leeks, asparagus, chicory, Jerusalem artichokes, turnips, beets, garlic, onions, eggplant, wheat, oats, whole grains, pears, peaches, plums, apples, oranges, kiwi, almonds, sesame seeds, flax seeds, and mushrooms [57,262,263,264].

Sources of fiber from food products and supplements and their therapeutic roles are presented in Table 3 and Table 4.

**Figure 6:**
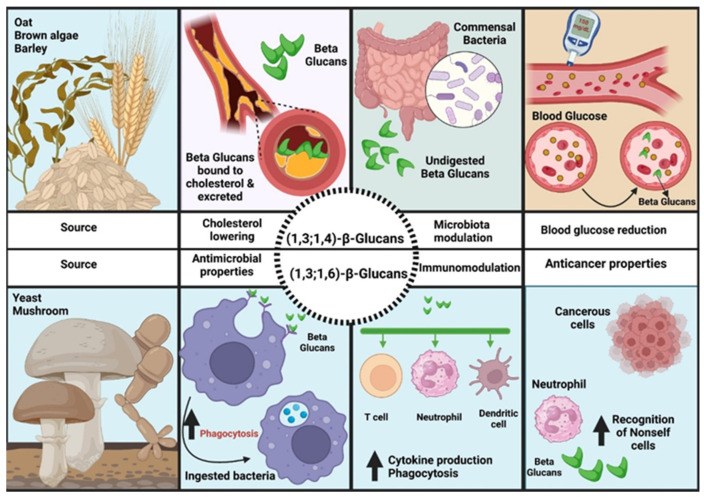
Sources of β-glucan and its effects on pathophysiological links. Created with BioRender.com (accessed on 15 November 2023).

Below, in Figure 6, various sources of β-glucan and their effects on pathophysiological links are shown. β-Glucans, classified as soluble fibers, are present in the cell walls of diverse organisms such as bacteria, fungi (including yeast and mushrooms), algae, and select plant species like oats and barley. They exhibit prebiotic properties, fostering the proliferation of advantageous gut bacteria. This amplifies the maintenance of a robust gut microbiome, which correlates with multifaceted facets of overall health, including gastrointestinal function and immune modulation [267,268].

Renowned for their extensive health advantages, β-glucans possess notable immunomodulatory effects. Their interaction with immune cells, notably macrophages, dendritic cells, and natural killer cells, augments their functionality. Consequently, this fortifies the body’s defenses against infections and contributes to managing specific autoimmune conditions by finely regulating immune responses.

Additionally, β-glucans manifest antioxidant characteristics, mitigating oxidative stress within the body. This pivotal function prevents cellular damage instigated by free radicals, which have associations with a spectrum of ailments encompassing cancer and cardiovascular maladies. Moreover, they exhibit anti-inflammatory properties by impeding select inflammatory pathways, potentially offering benefits in conditions where excessive inflammation contributes to disease progression [247,252].

Research indicates that β-glucans sourced from oats and barley, in particular, may contribute to the reduction in LDL (low-density lipoprotein) cholesterol levels. Their capacity to form a gel-like substance within the digestive tract facilitates the binding of cholesterol, impeding its absorption and thereby supporting cardiac health [247,266,267].

Moreover, β-glucans play a role in regulating blood sugar levels by retarding the absorption of sugars within the digestive system. This attribute is advantageous for individuals with diabetes or those predisposed to developing the condition.

In summary, β-glucans exert a profound influence on diverse physiological processes within the body, spanning immune modulation, inflammation mitigation, cholesterol regulation, blood sugar homeostasis, and gut health. Their multifaceted benefits underscore their significance in both preventive healthcare strategies and potential therapeutic applications.

To date, from the existing studies, a minimum dose of 3 g/day of β-glucan could be established for the cholesterol-lowering effect. A directly proportional dose-effect relationship could not be established; therefore, much higher consumption of β-glucan (>10 g/day) does not seem to show beneficial effects [177,265,280,281,282].

Also, no relationship of enhancement of effect could be established when treatment is continued for longer than 12 weeks. The effect of β-glucan on plasma cholesterol appears to remain stable over time. However, even though many types of fiber do not necessarily show a greater lowering effect in people with hypercholesterolemia, for β-glucan some studies show a greater reduction in cholesterol for people with high levels compared to healthy individuals. This enhanced cholesterol-lowering effect can also be seen in people with type 2 diabetes [247,265,280,281].

Although fiber-rich foods have a protective role on the gastrointestinal microbiome, modern man tends to eat processed food, rich in calories, fat, and sugars, and which does not offer any nutritional value. At the same time, food choices are influenced by a sedentary lifestyle, by advertisements with visually appetizing products, by social media, resulting in an alarming increase in the incidence of obesity in both adults and children.

A review published in 2021 [282] indicates the changes induced by junk food in the body but also the population of intestinal microbiota. Margarine, fries, doughnuts, ice cream, and baked goods contain many trans fats that induce metabolic changes, resulting in increased inflammatory markers, diabetes, cancer, and increased risk for cardiovascular disease. High-fructose corn syrup found in sodas and sweetened carbonated beverages is positively correlated with obesity, diabetes, high blood pressure, atherosclerosis, coronary heart disease, and renal vascular resistance. Mayonnaise and roast pork contain propyl gallate which has been associated with reproductive toxicity, testicular and abnormal placental development. Monosodium glutamate found in fish and soy sauces is considered a neurotoxic compound that can contribute to obesity, brain damage, diabetes, and endocrine disorders [282].

Furthermore, the intake of sugary foods is linked to an escalated risk of developing cardiovascular diseases, irrespective of overall caloric intake. Despite this well-documented knowledge, sugar consumption globally is on the increase. Heightened fructose consumption has been associated with numerous significant metabolic disorders, including dyslipidemia, insulin resistance, diabetes mellitus, hyperuricemia, and non-alcoholic fatty liver disease [283]. It is important to state the significant increase in the prevalence of these cardiometabolic diseases in the context of the recent pandemic situation that has spanned an entire year and has determined a series of changes in the lifestyle of modern people.

With the isolation due to the COVID-19 virus, the usual food pattern has been compromised. Food choices, correlated with the sedentary lifestyle have determined an increased incidence of various diseases, especially through the alteration of the immune system caused by junk food (many people have opted for snacks as comfort food, given the fact that food brings emotional comfort) [259,267,284]. At the same time, the fear of isolation and limiting contact with the outside led many people to buy in excess, to stock up, with a possible consumption of these foods excessively.

It is important to pay special attention to the conditions for obtaining and processing food products, the re-contamination of contaminants below the maximum allowed limits is crucial for consumer safety, food contaminants (pesticides, heavy metals, microplastics, pathogenic microorganisms) can significantly affect both the intestinal microbiome and the health of the whole organism, endangering even the consumer’s life [285,286,287,288]. The same care must be given to the raw materials used to obtain nutritional supplements or medicines related to the lack of potentially toxic contaminants [279,280,281,282,283,284,285,286,287,288,289,290,291,292].

Dietary fibers, thanks to their swelling properties, increase the viscosity and fecal volume, dilute carcinogens, and reduce the time of proteolytic fermentation, thus reducing the contact between carcinogens and the intestinal mucosa [258].

Fiber binds and excretes carcinogens from the intestinal lumen and changes the pH in the colon. They have the property of binding with useful phytochemicals such as carotenoids, arabinoxylan, flavonoids, and lignans, thus fortifying the antioxidant activity by reducing free radicals, forming bioactive complexes that protect the gastrointestinal tract from oxidative damage [258].

In the colon, dietary fibers contribute to the synthesis of SCFAs, which foster a healthier balance within the intestinal microbiota, also bolstering immunity and preventing neoplastic developments. SCFAs have been shown to interrupt the cancer cell cycle and restrain chronic inflammatory processes within the colon [258].

Recent studies have demonstrated the beneficial role of dietary fiber not only in preventing colorectal cancer but also in various other cancer types such as small bowel, laryngeal, and breast cancers. Although the precise mechanisms are not fully understood, several proposed modes of action include

- Resistance of dietary fiber to digestion in the small intestine, enabling its fermentation in the large intestine to generate short-chain fatty acids with known anticancer properties.
- Increase in fecal volume and viscosity due to dietary fiber, reducing the duration of contact between potential carcinogens and mucosal cells.
- Enhanced binding of bile acids to carcinogens facilitated by dietary fiber.
- Elevated levels of antioxidants associated with increased dietary fiber intake.
- Inhibition of estrogen absorption in the intestines leading to increased excretion of estrogen in feces by dietary fiber.

In terms of breast cancer, a prospective cohort study involving over 90,000 premenopausal women revealed that higher fiber intake, particularly during adolescence, reduced the risk of breast cancer. A comparison of the highest versus lowest fiber intake exhibited a 25% reduction in breast cancer risk [293].

Moreover, a subsequent meta-analysis encompassing 17 prospective cohort studies echoed the protective effect of high fiber intake against breast cancer risk, on both premenopausal and postmenopausal breast cancers [294]. Additionally, a high-fiber diet has been linked to a decreased risk of benign breast disease, recognized as a risk factor in adolescents for the later development of breast cancer [295]. Health specialists recommend to the general public a consumption of 14 g of fiber for every 1000 kcal consumed, distributed equally throughout the day, within each meal and/or snack. The required daily energy intake changes in relation to the age and gender of the individual, so the nutritionist estimates the amount of fiber needed by the individual depending on the gender and the age category in which they fall [296].

## 6. The Microbiome and Longevity

The microbiome, an assembly of numerous microorganisms primarily situated in the gastrointestinal tract, exerts significant influence over multiple facets of health and potentially affects longevity. A well-diversified and harmonized gut microbiome correlates with an efficient immune system, facilitating pathogen defense and immune regulation, thus potentially diminishing the incidence of chronic ailments that could compromise lifespan. Moreover, maintaining an equilibrated inflammatory response is facilitated by a healthy microbiome; microbial imbalances (dysbiosis) are implicated in chronic inflammation, a factor associated with various age-related maladies. Managing inflammation assumes paramount importance in sustaining overall health and longevity [297,298,299,300].

Beyond immune and inflammatory modulation, gut bacteria are instrumental in metabolizing dietary constituents and regulating energy metabolism. Imbalances within the microbiome can contribute to metabolic dysfunctions like obesity and diabetes, factors linked to reduced lifespan. Additionally, specific gut bacteria produce bioactive compounds, such as short-chain fatty acids (SCFAs) and vitamins, which contribute holistically to health. SCFAs, renowned for their anti-inflammatory properties, exhibit the potential to positively influence metabolic and immune functions.

Evidence supports a correlation between the gut microbiome and age-associated ailments like cardiovascular diseases, neurodegenerative disorders, and certain cancers. A balanced microbiome may serve to mitigate the risk or progression of these conditions, thereby potentially contributing to an extended lifespan. Moreover, gut microbes are intricately involved in nutrient breakdown and the synthesis of crucial compounds. An optimally functioning microbiome ensures efficient nutrient absorption, thereby contributing significantly to overall health and potentially impacting longevity.

Recent investigations hint at the influence of the gut microbiota on hormonal balance, thereby impacting processes like insulin sensitivity, which hold implications for metabolic health and the aging process [301,302,303].

While the relationship between the microbiome and longevity is increasingly elucidated, it is essential to acknowledge the multifaceted contributors to lifespan, including genetics, lifestyle choices, dietary habits, environmental influences, among others. Although interventions such as dietary modifications, probiotics, and lifestyle adjustments may hold promise in positively shaping health and potentially extending lifespan through microbiome modulation, further comprehensive research is imperative to establish definitive and comprehensive connections.

Everyone wishes to live a long and fulfilling life with their loved ones and uncover the secret of “eternal youth” Recently, researchers have extensively explored the impact of the microbiome on longevity through clinical trials using prebiotics and probiotics. Positive connections have been found between the ecosystem of bacteria in the gut and an individual’s lifespan, involving various intricate cellular mechanisms [304,305]. An important aspect regarding anti-aging effects is represented by the interactions occurring between *Bifidobacteria* in the gastrointestinal tract and butyrate-producing colonic bacteria (*Fecalibacterium prausnitzii* and *Roseburia*). Butyrate appears to play a crucial role in the aging process and age-related diseases (i.e., degenerative processes and pathologies) due to its involvement in modulating epigenetic processes through the inhibition of histone deacetylase activity [306]. An increased butyrate production is one of the indicators of gut health [307].

According to Élie Metchnikoff, Nobel Prize laureate in 1908, the most effective and accessible means to combat senescence is through a proper diet. Metchnikoff viewed aging and death as a form of intoxication that could be counteracted with appropriate nutrition. In his theory, fermented/acidified dairy products (such as yogurt) containing lactic acid bacteria were considered capable of suppressing “putrefactive” microflora and detoxifying the body. Metchnikoff himself adhered to this dietary recommendation and attributed his relatively high longevity to it, despite coming from a family with a very short life expectancy. Thus, in the pursuit of therapies and means to combat aging, Metchnikoff incorporated the science and industry of probiotics [308].

In the 21st century, there have been intense research efforts in the field of gerontology, particularly aimed at identifying anti-aging solutions [309].

With the increasing interest in this field, there has been progress that enhances our understanding of the role of the intestinal microbiota in human health, aging, and longevity. To modulate the activity of the intestinal microbiota and fully benefit from its positive effects on the body, it is important to consider factors that positively influence the bacterial community. These factors include caloric restriction through a nutrient-rich diet with specific energy intake tailored to the body’s needs, regular physical activity, the consumption of probiotics and prebiotics, fecal microbiota transplantation in the presence of existing infections or pathologies that disrupt the bacterial community balance, as well as environmental factors and mindfulness practices [310,311,312,313,314,315].

There are gaps in the literature regarding the influence of the biodiversity of microorganisms in the intestines on telomeres (essential structures located at the ends of chromosomes that prevent their degradation and/or fusion with other chromosomes).

Chen SS et al. (2022) conducted a cross-sectional study among 401 Chinese children aged 6–9 years to examine relationships between gut microbiota composition, fecal short-chain fatty acids (SCFAs), and leukocyte telomere length (TL) [316]. Extending the inquiry into the relationship between TL and gut diversity, specific operational taxonomic units (OTUs) of gut microbiota have been found to be negatively correlated with leukocyte TL significantly, thereby suggesting some correlation with cellular aging. However, the concentrations of fecal SCFAs did not reveal a significant correlation. This study suggests the importance of gut microbiota in influencing cellular aging processes in children [316].

The study by Ghosh TS et al. (2020) explored the impact of a Mediterranean diet (MedDiet) on the gut microbiome of 612 elderly individuals across five European countries, aiming to reduce frailty and improve health status over a year [317]. The findings revealed that adherence to the MedDiet led to significant changes in the gut microbiome, correlating with reduced frailty, improved cognitive function, and decreased inflammation markers. These outcomes were associated with increased production of beneficial SCFAs and a decrease in harmful secondary bile acids, highlighting the potential of dietary interventions to modulate the gut microbiota and promote healthier aging [317]. Even if the genetic component cannot be modified, the epigenetic is flexible. We are at a point in time where we have sufficient knowledge to understand that we can prevent chronic diseases and promote healthier aging by choosing what is at the end of our fork.

## 7. Fecal Microbiota Transplantation: A Novel Therapy in Dysbiosis-Related Diseases

Fecal microbiota transplantation (FMT), also known as fecal microbiota transplantation, is a therapeutic modality involving the transfer of fecal material from a healthy donor to an afflicted recipient to restore microbial equilibrium within the gastrointestinal tract. The procedure typically involves the meticulous selection and screening of a healthy donor, followed by the processing of fecal material to create a microbiota-rich suspension. Subsequently, this suspension is administered to the recipient, with delivery methods encompassing nasogastric/nasoduodenal/nasojejunal tube infusion, colonoscopy, or encapsulated formulations [318,319,320,321,322]. The protocol for preparing fecal samples to be delivered to the patient has been extensively presented in the literature [323,324,325]. Donors must meet strict eligibility criteria and undergo multiple investigations to eliminate exclusion factors. The exclusion criteria are as follows [326]: individuals younger than 18 years or older than 65 years, body mass index exceeding 30 kg/m2, significant undernutrition (moderate-severe) or the presence of metabolic syndrome, antibiotic treatment within the past 6 months, episodes of diarrhea in the past 3 to 6 months, previous infection with *Clostridium difficile*, recent factors increasing the risk for human immunodeficiency virus (HIV) or viral hepatitis, conditions related to immune dysfunction or the use of drugs that suppress the immune system (immunosuppressive medication), recent travel to tropical areas within the last 3 months, any gastrointestinal conditions (inflammatory bowel disease, irritable bowel syndrome, and others), autoimmune or atopic diseases, chronic pain disorders (fibromyalgia and/or chronic fatigue syndrome), neurological or developmental disorders, a history of cancer. These criteria are proposed for identifying suitable stool donors, though each institution may adapt these guidelines as per their specific requirements.

FMT has gained notable recognition for its efficacy in addressing recurrent *Clostridioides difficile* infection (CDI), often stemming from antibiotic-induced dysbiosis [327]. Through the administration of diverse and functional microbiota from a healthy donor, FMT effectively counters the pathological proliferation of *Clostridioides difficile*, thus addressing recurrent CDI [328,329].

While FMT has demonstrated success in the context of CDI, ongoing scientific investigations are exploring its potential applications in diverse pathological conditions associated with dysbiosis, including but not limited to inflammatory bowel diseases (IBD) represented by Crohn’s disease [330,331] and ulcerative colitis [321,332,333], irritable bowel syndrome (IBS) [322,334,335], and metabolic disorders [318,336,337,338]. The underlying premise is that restoration of a balanced gut microbiota may exert therapeutic effects beyond the scope of CDI. Numerous clinical studies have also achieved remarkable results in certain pathologies that are not limited to gastrointestinal damage, such as liver diseases [339,340,341], cardiovascular diseases, cancer, autoimmune diseases, infectious diseases, neurological and neurodegenerative disorders. According to Bajaj JS at al. (2021), FMT has been found to reduce alcohol craving (genera *Bilophila* and *Ruminococcus* were identified as contributing factors), improve cognitive function, and enhance overall quality of life [342].

Mocanu V et al. (2021) estimated the effect of fecal microbial transplantation (FMT) on dietary fiber supplementation among adult patients with obesity and metabolic syndrome [338]. The findings supported the perspective that FMT alone, but especially together with low fermentable fiber, might improve insulin sensitivity, modify microbial diversity, and potentially be used as a biotherapeutic for metabolic syndrome. Indeed, the study proved the tolerability of FMT and fiber supplements, as well as identified specific microbial taxa predictive of improved insulin sensitivity. The research enlightens that the metabolic improvements witnessed due to fecal microbial transplantation (FMT) and low fermentable fiber supplementation are a result of a comprehensive mechanism. Microcrystalline cellulose fiber acts as a bulking and binding agent which, in essence, increases the concentration of microbial metabolites within the gut. In this very spirit, it changes the kinetics of gastrointestinal transit and hosts the donor microbes differently with the mucosa layer. Such direct supplementation changes the role of some very specific microbial groups through increased efficiency in fermentation and production of its metabolic by-products. It contributes to increasing the diversity and richness of gut microbes and beneficial shifts in the microbial taxa, which have been known to enhance insulin sensitivity and alter indices of insulin resistance. Moreover, the metabolic benefits are related to enteroendocrine effects, which are responsible for gut hormone secretion regulating glucose metabolism, and insulin sensitivity. In addition, post-FMT dietary interventions, especially low-fermentable fiber, favor the engraftment of beneficial donor microbes to improve metabolic health in individuals with severe obesity and metabolic syndrome [338].

In a trial conducted by Allegretti JR et al. (2021) on FMT in obese, metabolically healthy patients, the study suggested the potential to prevent metabolic syndrome [319]. There were 22 participants in this trial with great improvement in insulin and glucose at 6 and 12 weeks after FMT compared to placebo. In particular, glucose AUC was significantly reduced by week 12 (579 vs. 1978, *p* = 0.03) and insulin AUC by week 6 (137 vs. 2728, *p* = 0.01). These findings provide promising evidence in favor of FMT influencing metabolic health and argue for further studies, as the obesity epidemic drastically limits effective medical therapy options [319]. Conversely, Yu EW et al. (2020) observed no significant improvements in insulin sensitivity or metabolic parameters despite successful gut microbiota engraftment, indicating the complexity of FMT’s effects on metabolism [320]. These conflicting results could be due to differences in study design, participant selection, and methods of FMT administration. Allegretti et al. focused on metabolically healthy obese individuals, possibly capturing early preventive effects [319]. Yu et al.’s participants had mild to moderate insulin resistance, possibly affecting the FMT’s impact [320]. Additionally, variations in donor microbiota, FMT preparation, and the recipients’ baseline microbiome diversity could influence outcomes. Future research should explore these variables to optimize FMT’s therapeutic potential.

Despite the benefits of this procedure, some numerous gaps and limitations indicate caution concerning FMT. While FMT is generally considered safe, there are concerns about the potential transmission of infectious agents and the long-term risks associated with altering the gut microbiome [343]. Also, the mechanisms through which FMT influences disease processes, including its effects on immune modulation, metabolic pathways, and neurobehavioral outcomes, are not fully understood. In general, the gut microbiota is capable of communication with distant organs via the gut–brain axis, gut–liver axis, and gut–heart axis among others [344,345]. Therefore, the effects of gut microbiota dysbiosis on the host would be expressed through the modulation of the endocrine and immune systems [344]. This research gap hinders the development of targeted microbiota therapies that could enhance FMT’s efficacy across different conditions. Adverse effects associated with FMT predominantly stem from insufficient scrutiny of fecal donor material and the potential aggravation of chronic conditions in recipients [346]. This highlights the critical need for rigorous donor screening and careful patient selection to mitigate risks associated with FMT procedures.

It is essential to emphasize that FMT is a medical intervention requiring strict adherence to safety protocols. Donors undergo comprehensive screening to ensure the absence of infectious agents, and the procedure itself demands careful clinical supervision. FMT has promising therapeutic potential, but it also carries potential risks and negative effects, which highlights the need for judicious application guided by current scientific evidence and under the purview of healthcare professionals.

Additionally, the significant recent developments in the field of fecal microbiota transplantation (FMT) therapy for *Clostridioides difficile* (*C. difficile*) infection must beighlightedd. In 2022, the U.S. Food and Drug Administration (FDA) approved FMT therapy for the treatment of severe *C. difficile* infection, marking a significant milestone in the recognition of FMT as an effective therapeutic option [347]. This approval encompasses both traditional colonoscopic administration and the first orally administered FMT product, providing patients with additional treatment options and expanding access to this potentially life-saving therapy [348].

Furthermore, the adoption of FMT as routine treatment for *C. difficile* infection has gained traction in European hospitals. For example, the Microbiome Unit at Bellvitge University Hospital in the European Union has incorporated FMT into its treatment protocols, underscoring the growing acceptance and utilization of FMT as a standard-of-care therapy for *C. difficile* infection [349]. These developments represent important advancements in microbiome-based therapeutics and have significant implications for clinical practice.

## 8. Conclusions

Lifestyle and food quality significantly influence the balance of the gut microbiome. Thus, processed food, fast food, refined sweets, sweetened carbonated juices, and sauces negatively influence the intestinal microbiome by altering its composition (dysbiosis). The consumption of these foods harmful to the body is becoming more and more alarming, despite the warnings of organizations in the field. These foods have a negative impact on the saprophytic intestinal microbiota with the reduction in beneficial bacterial species and the favoring of opportunistic or harmful genera. Changes in the gut microbiome have been linked to various disorders affecting the digestive, cardiovascular, nervous, immune, muscle, and skeletal systems. This means that changing the intestinal microbial composition has an impact on the entire body, so food choices play a fundamental role in maintaining health (which is not exclusively the absence of disease).

Removing harmful foods rich in saturated fat, trans fat, sugars, salt, and artificial sweeteners from the market would clearly limit their consumption and limit non-communicable chronic diseases.

By introducing a diverse array of microbial species, FMT seeks to reestablish a resilient and functional gut microbiome, thereby alleviating symptoms associated with conditions such as inflammatory bowel disease (IBD), Clostridioides difficile infection (CDI), and irritable bowel syndrome (IBS). Through a process of microbial engraftment and ecological restoration, FMT holds the potential to modulate host immune responses, enhance metabolic functions, and promote intestinal barrier integrity. While further research is warranted to optimize FMT protocols and elucidate their long-term effects, the therapeutic promise of this approach underscores its significance as a transformative strategy in the management of dysbiosis-related disorders.

## Figures and Tables

**Table 2:** Pathologies associated with dysbiosis and changes detected at the level of intestinal microbial communities.

Pathology	Disease Type	Changes in the Microbiota	References
Obesity	Metabolic disease	↑ Increased levels: *Bacillota phylum:Bacteroides* *Methanobrevibacter smithii* *Lactobacillus* (*Lactobacillus reuteri*) *Desulfovibrionaceae* ↓ Decreased levels: *Bifidobacteria* *Escherichia coli* *Akkermansia muciniphila*	[206,207,208,209,210,211,212]
Severe acute malnutrition (SAM)	Metabolic disease	↑ Increased levels: *Proteobacteria* *Bacteroidaceae* *Porphyromonadaceae* *Bilophila* *Klebsiella* *Escherichia coli* *Streptococcus* *Shigella* *Enterobacter* *Veillonella* ↓ Decreased levels: *Bacteroidetes* *Roseburia* *Fecalibacterium* *Butyribrio* *Synergistetes* *Methanobrevibacter smithii*	[213,214,215]
Type 2 diabetes (T2D)	Metabolic disease	↑ Increased levels: *Bacteroidetes-Bacillota phylum* *Bacteroidetes-Prevotella* *Betaproteobacteria* *Clostridium* spp. *Bacteroides caccae* *Desulfovibrionaceae* spp. ↓ Decreased levels: *Roseburia* spp. *Bacillota phylum* *Clostridia*	[216,217]
Metabolic syndrome	Cardiometabolic disease	↑ Increased levels: raport *Bacillota phylum:Bacteroides* *Methanobrevibacter smithii* *Lactobacillus* (*Lactobacillus reuteri*) ↓ Decreased levels: *Bifidobacteria* *Escherichia coli*	[211,216,217,218]
Ischemic or dilated cardiomyopathy	Cardiovascular disease	↑ Increased levels: *Prevotella* *Hungatella* (*Lacnospiraceae*) *Succiniclasticum* *Ruminococcus* *Acinetobacter* *Veillonella* ↓ Decreased levels: *Blautia* *Anaerostipes* *Fecalibacterium* *Lachnospiraceae* *Bifidobacterium* *Eubacterium* *Coprococcus* *Alistipes* *Oscilibacter*	[219,220]
Heart failure with reduced ejection fraction	Cardiovascular disease	↑ Increased levels: *Streptococcus* *Veillonella* *Eggerthela* ↓ Decreased levels: *Prevotella* *SMB53* (*Clostridiaceae*)	[221]
Coronary artery disease	Cardiovascular disease	↑ Increased levels: *Escherichia-Shigella* *Lactobacillus* *Enterococcus* *Streptococcus* ↓ Decreased levels: *Fecalibacterium* *Roseburia* *Eubacterium* *Subdoligranulum*	[222]
Hypertension	Cardiovascular disease	↑ Increased levels: *Klebsiella* *Salmonella* *Streptococcus* *Clostridium* *Parabacteroides* *Eggerthella* *Prevotella* *Porphyromonas* ↓ Decreased levels: *Fecalibacterium* *Roseburia* *Synergistetes* *Bifidobacterium* *Oscillibacter* *Coproroccus* *Butyrivibrio*	[223,224]
Atherosclerosis with clinical presentation of stable or unstable angina or myocardial infarction	Cardiovascular disease	↑ Increased levels: *Enterobacteriaceae* *Streptococcus* *Lactobacillus salivarius* *Atopobium parvulum* *Ruminococcus gnavus* *Eggerthella lenta* ↓ Decreased levels: *Roseburia* *Fecalibacterium*	[225]
Irritable bowel syndrome (IBS)	Gastrointestinal disease	↑ Increased levels: *Bacillota phylum:Bacteroides* *Proteobacteria* (*Enterobacteriaceae* spp.) *Bacillota phylum* *Lachnospiraceae* *Veillonella* *Streptococci* *Ruminococcus* spp. ↓ Decreased levels: *Lactobacillus* *Actinobacteria* (*Bifidobacteria*, *Colinsella*) *Bacteroidetes* *Fecalibacterium*	[226,227,228,229]
Inflammatory bowel disease (IBD)	Gastrointestinal disease	↑ Increased levels: *Proteobacteria* ↓ Decreased levels: *Lachnospiraceae* *Bacteroidetes*	[31]
Crohn’s disease (CRD)	Gastrointestinal disease	↑ Increased levels: *Roseburia hominis* *Ruminococcus gnavus* ↓ Decreased levels: uncharacterized species of *Clostridium* spp. *Fecalibacterium prausnitzii* *Bifidobacterium adolescentis* *Dialister invisus*	[34,118]
Ulcerative colitis (UC)	Gastrointestinal disease	↓ Decreased levels: *Roseburia hominis* *Fecalibacterium prausnitzii*	[230]
Celiac disease (CD)	Gastrointestinal disease	↑ Increased levels: *Bacteroides* *E.coli* ↓ Decreased levels: *Bifidobacterium longum* *Clostridium histolyticum* *C. lituseburense* *Fecalibacterium prausnitzii*	[119,120,121]
Colorectal cancer (CRC)	Gastrointestinal disease	↑ Increased levels: *Bacteroides fragilis* *Enterococcus* *Escherichia/Shigella* *Klebsiella* *Streptococcus* *Peptostreptococcus* *Dorea* spp. *Fecalibacterium* spp. *Fusobacterium* spp. ↓ Decreased levels: *Roseburia* *Lachnospiraceae* *Bacteroides* spp. *Coprococcus* spp.	[123,124,231]
Viral hepatitis	Liver disease	↑ Increased levels: *Enterobacteriaceae* *Enterococcus fecalis* *Escherichia coli* *Fecalibacterium prausnitzii* ↓ Decreased levels: *Leuconostoc* *Lactobacillus* *Weissella* *Pediococcus*	[232,233]
Liver cirrhosis secondary to hepatitis B or C virus infection (HBV/HCV)	Liver disease	↑ Increased levels: *Enterobacteriaceae* (*Neisseria*, *Gemella*) *E. Fecalis* *E. coli* *F. prausnitzii* *Candida* *Veillonella* *Megasphaera* *Dialister* *Atopobium* *Prevotella* ↓ Decreased levels: *Bacteroidaceae* *Ruminococcaceae* *Lachnospiraceae* *Bacillota phylum*	[233,234,235,236,237]
Anorexia nervosa (AN)	Mental illness	↑ Increased levels: *Methanobrevibacter smithii* *Bacillota phylum* *Actinobacteria* *Bacteroidetes* ↓ Decreased levels: *Lactobacillus plantarum* *Streptococcus* spp. *Clostridium coccoides* *Bacteroides fragilis*	[112,113,238,239]
Autism spectrum disorders (ASD)	Neurodevelopmental disability	↑ Increased levels: raport *Bacillota phylum-Bacteroidetes* *Sutterella* *Lactobacillus* *Clostridium* *Bacteroidetes* *Desulfovibrio* *Caloramator* *Sarcina* ↓ Decreased levels: *Bifidobacterium* spp. *Bacillota phylum* *Akkermansia muciniphila*	[240,241,242,243,244]
Alzheimer’s diseases (AD)	Neurodegenerative disorder	↑ Increased levels: *Escherichia/Shigella* *Bacteroidetes* ↓ Decreased levels: *E. rectale* *Bacillota phylum* *Bifidobacterium*	[245,246]
Parkinson’s disease (PD)	Neurodegenerative disorder	↑ Increased levels: *Lactobacillaceae* *Barnesiellaceae* *Enterococcaceae* *Ruminococcaceae* *Akkermansia* ↓ Decreased levels: *Lachnospiraceae*	[247,248]
Stress	Risk factors	↑ Increased levels: *Clostridium* spp. *Enterobacteriaceae* *Escherichia coli* *Pseudomonas* spp. ↓ Decreased levels: *Bacteroides* spp. *Lactobacilli* spp.	[249,250]

↑—increase, ↓—decrease.

**Table 3:** Food products and supplements and their therapeutic roles observed in human studies.

Food Product/Supplements	Subjects	Type of Subject	Study Design	Dose/Quantity Administered and Duration of Treatment	Recorded Effects	References
Oat β-glucan	Obese subjects with type 2 diabetes (n = 37; female = 28, male = 9)	Human	Randomized, double-blind, clinical trial Taken as a supplement, daily, with water or milk at room temperature No changes in the subject’s diet	5 g/day × 12 weeks	Reduction in HbA1c, C peptide; lowering of total cholesterol, VLDL-cholesterol and triglycerides; increased leptin levels decreased GLP-1 (glucagon-like peptide); increase in PYY; decreased populations of *Lactobacillus* spp. and *Bifidobacterium* spp.; improvement of intestinal transit	[265]
Concentrated oat β-glucan (54%)	Patients with hypercholesterolemia (n = 75; female = 50, male = 25)	Human	Randomized, double-blind, clinical trial Taken as a supplement No changes in the subject’s diet	6 g/day × 6 weeks	Reduction in total cholesterol and LDL-cholesterol; the treatment group reported increased flatulence as a symptom; increased production of SCFAs (especially butyrate)	[266]
Oligofructose (OFS)	Adults with obesity (n = 39; female = 32; male = 7)	Human	Randomized, double-blind, clinical trial No specific dietary instructions	21 g/day × 12 weeks	Weight loss; glucose levels decreased in the group receiving OFS and increased in the control group	[267]
Chitosan oligosaccharides (COS)	Patients with coronary artery disease/CAD (n = 120; female = 61; male = 49)	Human	Randomized clinical trial Hospital diet and normal control diet	2 g/day × 6 months	Reduction in serum levels of triglycerides, total cholesterol, LDL-cholesterol; decreased levels of transaminases ALT and AST; increase in HDL-cholesterol level Increase levels of antioxidants SOD and GSH; increased bacterial levels of *Bacteroides*, *Megasphaera*, *Roseburia*, *Prevotella*, *Bifidobacterium*; decreased bacterial levels of *Fecalibacterium alistipes*, *Escherichia coli*	[268]
Soluble fiber from corn (SCF)	Boys (n = 15): 13–15 years Girls (n = 9): 12–14 years	Human	Randomized, double-blind, clinical trial Foods usually consumed by teenagers (hamburgers, sandwiches, potato chips and spaghetti). Diet composition (53% carbohydrates, 14% proteins, 33% fats, 15 g of fibers)	12 g SCF/day × 21 days	Increase in intestinal calcium absorption; decrease in the proportion of the *Bacillota phylum phylum*; increase in proportion of *Bacteroidetes phylum*	[269]
SCF	Postmenopausal women (n = 12)	Human	Randomized, double-blind, cross-over clinical trial SCF in muffins and fruit-flavored drinks No specific dietary instructions, but the subjects were asked to complete a 4-day diet record	10–20 g SCF/day × 6 months	Increased calcium retention in bones in postmenopausal women	[270,271]
Inulin	Patients with chronic kidney disease/CKD (n = 41; female = 16 male = 25)	Human	Prospective, case-control study Personalized low-protein diet, providing 30–35 kcal/kg/day	19 g/day × 6 months	Decreased levels of uric acid, serum insulin, glucose, HOMA-IR, CRP, homocysteine, total cholesterol and triglycerides; increase HDL-cholesterol	[272]
Inulin	Women with obesity and major depressive disorder (n = 45)	Human	Randomized, double-blind, clinical trial 25% calorie-restricted diet	10 g/day × 8 weeks	Enhancing the beneficial effects of a calorie-restricted diet on adipose tissue and total cholesterol levels	[273]
Inulin (Fibruline Instant)	Women (n = 32): 18–40 years	Human	Randomized, double-blind, cross-over clinical trial Inulin (dissolved in water) was consumed at breakfast, lunch and dinner Test meals (in the morning and 3 h later) with moderate iron bioavailability (cooked rice 50 g dry weight; pureed, boiled vegetable sauce 25 g fresh weight boiled for 4 h)	20 g/day × 4 weeks	Increasing the concentration of *Bifidobacteria*; decreased fecal pH	[274,275]
Yacon	Women with obesity (n = 26)	Human	Randomized double-blind clinical trial Energy-restricted diet (minus 500 kcal/day)	25 g/day × 6 weeks	Abdominal discomfort, abdominal pain, and flatulence were observed in the first few days; increasing the antioxidant capacity of plasma; decrease in concentration (protein carbonyl)	[276]

**Table 4:** Food products and supplements and their therapeutic roles observed in animal studies.

Food Product/Supplements	Subjects	Type of Subject	Study Design	Dose/Quantity Administered and Duration of Treatment	Recorded Effects	References
Larch Arabinogalactan (LAG)	Eight-week-old male Sprague Dawley/S	Rat lab	Randomized controlled trial LAG diet + Basal diet	50 mg/kg/day × 3 days	Reduction in Gelsolin gene and hif1-α gene expression, apoptotic cells, and p38 phosphorylation	[269]
Apple pectin (AP)	Eight-week-old male Sprague Dawley/SD (n = 36)	Rat lab	Randomized controlled trial AP diet for the experimental group and basal diet for the control group	10,40,100 and 400 mg/kg/day × 3 days	Intake of 100 and 400 mg/kg/day of apple pectin reduced infarct size (SI), defined as the ratio of infarct area (IA)/area at risk (AAR), compared to the group receiving an intake of 10 and 40 mg/kg/day; the intake of 100 mg/kg/day reduced the apoptosis process in experimentally induced infarct areas; the intake of 100 mg/kg/day registered a reduction in cleaved caspase-3, which induces apoptosis; the intake of 100 mg/kg/day increased the expression of Bcl-2, but decreased the expression of Bax, compared to the control group; the Bcl-2/Bax ratio (determinant of cell death or survival) was increased in the 100 mg/kg/day group	[275]
Garlic	5 weeks of age male C57BL/6N mice (n = 30)	Rat lab	Randomized clinical trial Normal diet (54% carbohydrate, 21% protein, 6% fat) and high-fat diet (10% carbohydrate, 21% protein, 40% fat)	5% in the diet for 12 weeks	Reduction in serum levels of GOT and GPT, total cholesterol, triglycerides and LDL, insulin, HOMA-IR; amelioration of high-fat diet-induced dyslipidemia Improved ratio (villus height/crypt depth) Reducing the concentration of branched-chain fatty acids (BCFAs); increasing abundance of *Lachnospiraceae*; decrease in *Prevotella* abundance	[277]
Amylosucrase-modified chestnut starch	Eight-week-old male C57BL/6N mice	Rat lab	Randomized controlled trial High-fat diet (45% fat of total energy)	1500 mg/kg bw	Decrease in body weight, white adipose tissue (upregulating SCFAs-GPR43-mediated pathway)	[278]
α-cyclodextrin (α-CD)	5-week-old male C57BL/6JJmsSlc mice (n = 15)	Rat lab	Randomized controlled trial High-fat diet	5.5% in the diet for 16 weeks	Decrease in body weight and adipose tissue; normalization of adipocyte sizes in epididymal adipose tissue; lowering blood glucose levels; increasing concentrations of *Lactobacillales*, *Bacteroides*; decreased abundance for *Clostridium*; increase in total SCFA content of the cecum; increase in lactic acid levels	[279]

## Data Availability

Not applicable.
